# Ecophysiology with barley *eceriferum* (*cer*) mutants: the effects of humidity and wax crystal structure on yield and vegetative parameters

**DOI:** 10.1093/aob/mcaa086

**Published:** 2020-05-03

**Authors:** Penny von Wettstein-Knowles

**Affiliations:** Department of Biology, University of Copenhagen, Ole Maaloees Vej, Copenhagen N, Denmark

**Keywords:** *Eceriferum* (*Cer*) genes, ecophysiology, epicuticular wax structure, *Hordeum vulgare* (barley), phyllosphere humidity, kernel yield, plant cuticle, hydrophilic domains, nutrient leaching, water loss

## Abstract

**Background and Aims:**

In addition to preventing water loss, plant cuticles must also regulate nutrient loss via leaching. The *eceriferum* mutants in *Hordeum vulgare* (barley) potentially influence these functions by altering epicuticular wax structure and composition.

**Methods:**

Cultivar ‘Bonus’ and five of its *cer* mutants were grown under optimal conditions for vegetative growth and maturation, and nine traits were measured. Nutrient and water amounts going through the soil and the amount of simulated rain as deionized water, affecting phyllosphere humidity, delivered during either the vegetative or maturation phase, were varied. *Cer* leaf genes and three wilty (*wlt*) mutations were characterized for reaction to toluidine blue and the rate of non-stomatal water loss.

**Key Results:**

Vegetative phase rain on ‘Bonus’ significantly decreased kernel weight and numbers by 15–30 %, while in *cer.j59* and *.c36* decreases of up to 42 % occurred. Maturation phase findings corroborated those from the vegetative phase. Significant pleiotropic effects were identified: *cer.j59* decreased culm and spike length and 1000-kernel weight, *.c36* decreased kernel number and weight, *.i16* decreased spike length and *.e8* increased culm height. Excepting *Cer.zv* and *.ym* mutations, none of the other 27 *Cer* leaf genes or *wlt* mutations played significant roles, if any, in preventing water loss. *Cer.zv* and *.ym* mutants lost non-stomatal water 13.5 times faster than those of *Cer.j*, *.yi*, *.ys* and *.zp* and 18.3 times faster than those of four cultivars and the mutants tested here.

**Conclusions:**

Using yield to measure the net effect of phyllosphere humidity and wax crystal structure revealed that the former is far more important than the latter. The amenable experimental setup described here can be used to delve deeper. Significant pleiotropic effects were identified for mutations in four *Cer* genes, of which one is known to participate in wax biosynthesis. Twenty-seven *Cer* leaf genes and three *wlt* mutations have little if any effect on water loss.

## INTRODUCTION

The predominant driving force for studying Gramineae cuticles has been their role in water loss. Less attention has been paid to water sorption or transport of metabolites across this hydrophobic barrier. Water is retained on smooth cuticle surfaces (Neinhuis and Barthlott, 1997), potentially contributing to leaching of nutrients from within the leaf. One attribute contributing to roughness of cuticle surfaces is the presence of epicuticular wax crystals. Wild-type barley leaves are densely covered with small lobed, crystalline wax plates consisting predominantly of primary alcohols (C_26_), while the uppermost internodes and leaf sheaths and spikes bear a dense array of very long thin, β-diketone crystalline tubes, predominantly C_31_ 14,16-dione (von Wettstein-Knowles, 2016). Crystal reduction modifies refraction of light from cuticle surfaces so that they no longer appear dull green but bright green, while the uppermost internodes, leaf sheaths and spikes are no longer blue but a bright green. Such phenotypic changes from glaucous to non-glaucous led initially to the identification of 79 *Eceriferum* (*Cer*) genes with a total of 1560 mutations in barley (Lundqvist and Lundqvist, 1988) that can be used to investigate the relationship between epicuticular waxes and leaching. Twenty-five of the *Cer* genes affected only leaf blades, 27 only the uppermost internodes and leaf sheaths plus spikes and 23 only spikes, while four affected all organs. Recently, mutations of two of the latter, *Cer.zv* and .*ym*, have been localized to the same gene, a GDSL-motif esterase/acyltransferase/lipase (Li *et al.*, 2017) indispensable for synthesis of the cuticle’s structural component, the cutin polymer, which, together with its embedded waxes, is required for water retention. Thus, 28 of the *Cer* genes affect leaf blades and are referred to hereafter as barley *Cer* leaf genes.

Four additional barley *Cer* genes have been cloned. The *Cer-cqu* gene cluster encodes polyketide diketone synthase [DKS, a β-ketoacyl-CoA-synthase (KCS) type III], a lipase/carboxyl transferase and a P450 hydroxylase, which are key players in the DKS pathway, synthesizing the β-diketone aliphatics (Hen-Avivi *et al.*, 2016; Schneider *et al.*, 2016; von Wettstein-Knowles, 2017). *Cer-zh* encodes a type I KCS that elongates the C_16_–C_20_ acyl chains required for synthesis of the primary alcohols (Li *et al.*, 2018).

Contact angles are a method for quantitating the roughness of cuticle surfaces (Holloway, 1969; Nienhuis and Barthlott, 1997). The *cer.j59* mutation, for example, reduces the contact angle from ~140 to 116° (17 %) on leaf blades (Zabka *et al.*, 2008) so that water drops no longer run off the surface. Other mutations resulting in the absence of the long thin tubes on wheat leaf sheaths and barley lemmas reduced contact angles by 14 and 20 %, respectively (Netting and von Wettstein-Knowles, 1973; King and von Wettstein-Knowles, 2000). An increase in moisture provides not only an atmosphere suitable for insect colonization and microorganism germination (Shepherd and Griffiths, 2006), but also in-ear sprouting of kernels (King and von Wettstein-Knowles, 2000) and leaching of metabolites, both organic and inorganic, through the cuticular layer of the apoplast into the phyllosphere (Tukey, 1970). Among potential roles for epicuticular waxes that have been probed with the *cer* mutants are the relationships of its structure, amount and/or composition with (1) powdery mildew and brown rust infection (Yang and Ellingboe, 1972; Rubiales *et al.*, 2001; Zabka *et al.*, 2008; Hansjakob *et al*., 2011; Li *et al.*, 2018), (2) attachment of herbivorous insects and their predators (Eigenbrode, 2004; Rostás *et al.*, 2008; Northfield *et al.*, 2012), (3) reflectance of electromagnetic radiation (Baker, 1974; Johnson *et al.*, 1983; Febrero *et al.*, 1998; Shepherd and Griffiths, 2006) and (4) yield (Gustafsson, 1963; Gustafsson *et al.*, 1975; Baenziger *et al.*, 1983; Johnson *et al.*, 1983; Febrero and Araus, 1994; Febrero *et al.*, 1998; Merah *et al.*, 2000; Monneveux *et al.*, 2004; Simmonds *et al.*, 2008).

In some environments glaucous genotypes with their rough leaf surfaces yield better than non-glaucous ones with smooth surfaces. Among these are several barley *cer* leaf mutants grown in dry environments (Baenziger *et al.*, 1983; Febrero *et al.*, 1998). While this difference was lost when *cer.j59* was grown under optimal conditions (Gustafsson, 1963), both *cer.j59* and *gl4* yielded less when grown under irrigated field conditions. By comparison, mutants affecting the uppermost leaf sheaths and exposed internodes plus spikes exhibit a wide range of yield phenotypes. Thus, compared with their mother varieties, *cer.c36* yielded 10–18 % less under favourable conditions (Gustafsson *et al.*, 1975), while another barley mutant, *gs2* (an allele of the *Cer.b* gene), grown under various field conditions had the same yield (McProud, 1971; Baenziger *et al.*, 1983). The decreased yield in dry environments was initially attributed to intracuticular waxes as water vapour would quickly diffuse through epicuticular wax crystals. Subsequently evidence was presented that the higher yields of glaucous lines were primarily attributable to decreased transpiration accompanied by a temperature reduction of photosynthesizing tissues and leaf senescence (Richards *et al.*, 1986). More recent studies have exploited the sensitive carbon isotope discrimination assay, the results of which are often closely correlated with yield (Farquhar *et al.*, 1989; Febrero *et al.*, 1998; Merah *et al.*, 2000; Monneveux *et al.*, 2004; Richards *et al.*, 2010). Water molecules are believed to diffuse across cuticles primarily via the lipophilic pathway, i.e. the amorphous phase of cuticular waxes (Riederer and Schreiber, 2001). But the contribution of intracuticular versus epicuticular waxes to transpiration is still a matter of debate. In deciduous leaves results imply that only intracuticular waxes are important, whereas for fruit cuticles epicuticular wax also plays a role (Kerstiens, 2006; Zeisler-Diehl *et al.*, 2018). In *Arabidopsis* leaves epicuticular wax was twice as effective as intracuticular wax in preventing water loss (Buschhaus and Jetter, 2012). These considerations reveal that questions remain about the molecular mechanism(s) in herbaceous species contributing to the described glaucosity/yield correlations.

To date no studies have been carried out on the potential effects of wax crystal structure on yield when moisture is in excess, e.g. in rainy environments. This is of interest given the early deduction that the amount of metabolites leaching from plants was related to hydrophobicity of the cuticle surface based on evidence from comparisons during development of a plant and between plants (Tukey, 1970). Here, ‘Bonus’ barley and its mutants *cer.j59*, .*c36*, .*u69,* .*i16* and *.e8* are exploited because of their known effects on wax structure to explore the contribution of the apoplast’s outermost layer to leaching, and the effect of humidity on this. The effects of delivering deionized water as simulated rain and in pertinent amounts through the soil during the vegetative and maturation phases on nine traits are investigated. Using yield parameters to measure leaching, results reveal that while this phenomenon can be modified by epicuticular wax structure, phyllosphere humidity is more important. Pleiotropic effects of *cer.j59*, .*c36*, .*i16* and .*e8* are identified and characterized, and the composition of the waxes on *cer.i16* and *.e8* spikes determined. The barley leaf *Cer* gene collection as well as three *wilty* (*wlt*) mutants, whose name suggests a defect in water retention, were characterized with respect to non-stomatal water loss rate and toluidine blue staining.

## MATERIALS AND METHODS

### Simulated rain experiments

**Fig. 1.**
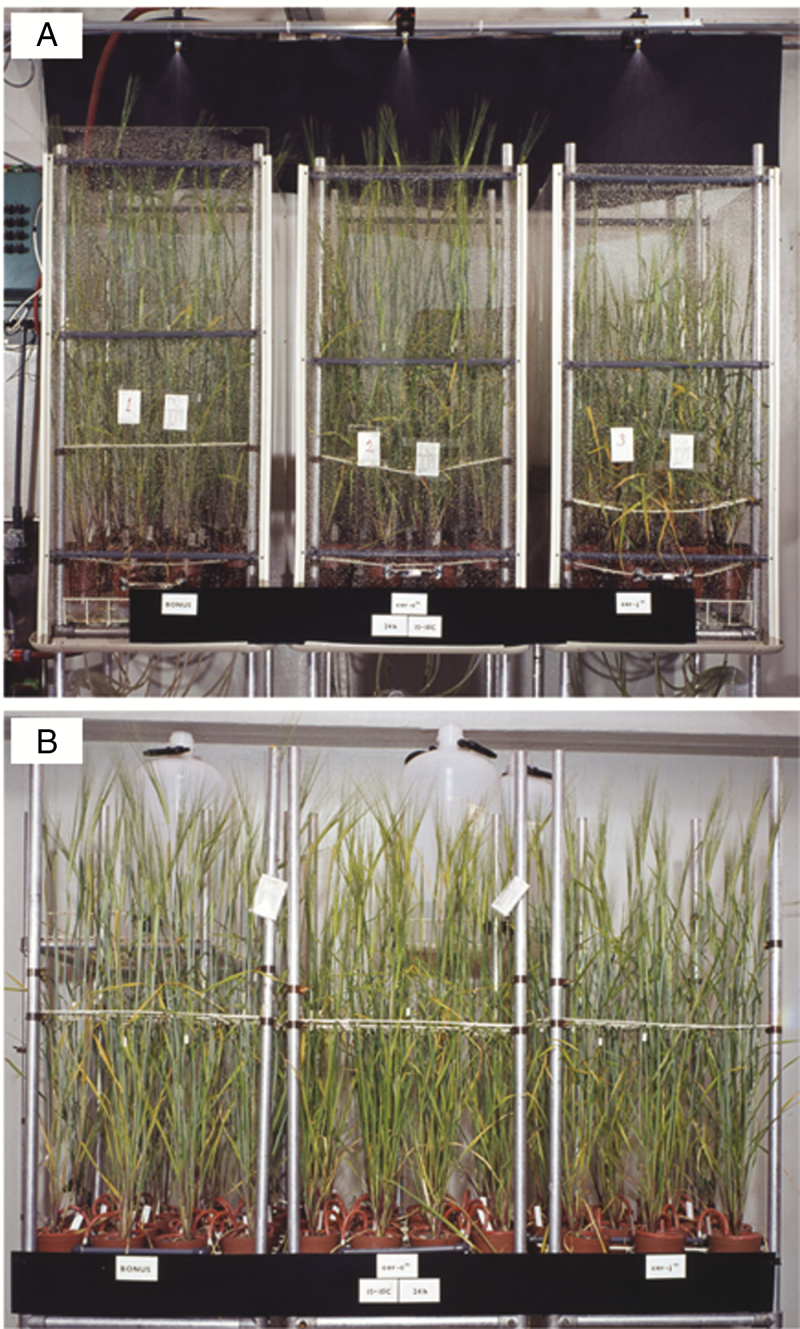
Trucks of ‘Bonus’ (left), *cer.c36* (centre) and *ce*r*.j59* (right) 57-d-old plants growing in the Stockholm phytotron under constant light and 15/10 °C (16/8 h) with nutrients every other day (1N) and 19.7 L water d^−1^ (10W) via (A) rain and (B) drip methods.

**Fig. 2.**
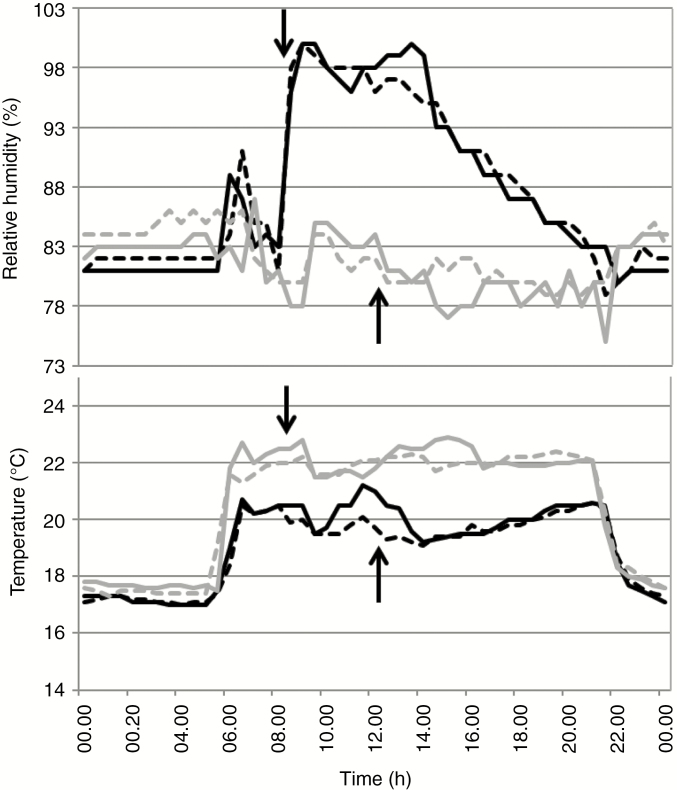
Relative humidity and temperature on two successive days in ‘Bonus’ trucks watered with 19.7 L water d^−1^ (10W) via rain and drip methods. Downward and upward arrows indicate the start and end of water supply, respectively. Solid black lines, rain day 1; dashed black lines, rain day 2; solid grey lines, drip day 1; dashed grey lines, drip day 2.

*Plant material and growth conditions*. Seeds of *Hordeum vulgare* ‘Bonus’ and the *eceriferum* mutants induced in this cultivar, *cer.c36*, *.e8*, .*i16*, *.j59* and .*u69*, were planted and grown in a mixture of sterilized sand and gravel in the Stockholm phytotron (von Wettstein, 1967) under optimum conditions for vegetative growth and seed maturation (Dormling *et al*., 1966, 1969). That is, they were under constant light for 60 d at 15/10 °C (16/8 h) during the vegetative phase, before being shifted to 23/18 °C (16/8 h) for the maturation phase. Fourteen 10.5-cm pots were present on each moveable truck. Besides the standard phytotron methods of watering using deionized water and giving nutrients either every other day (1N) or every day (2N), two other methods were employed. Parameters are specified in Tables 1 and 2 or given below. (1) Simulated rain, hereafter referred to as rain, illustrated in Fig. 1A: water was dispersed in varying amounts as detailed below for 4 h above the plants in cycles (Table 1) as a fine spray from a nozzle followed by a pause. The sides of the rain trucks from just above soil level were enclosed in plastic. (2) Drip, illustrated in Fig. 1B: an amount of water equivalent to that given as rain the previous day was dripped at pot level during the same 4 h from large storage tanks at a higher elevation. Water from the storage tank entered a pipe encircling the four central pots and was distributed from there individually through red tubing (Fig. 1A) to each pot. Drip truck sides were not enclosed. All water passing through the soil of each pot on both rain and drip trucks was collected (for example, see rubber tubing beneath pots on rain trucks in Fig. 1A) and measured. Figure 2 illustrates the temperature and relative humidity on both a rain and a drip truck of ‘Bonus’ measured in the truck centre every half-hour for two successive days during the maturation phase. While the thermoperiod of the drip truck was ~22/17–18 °C within the optimum range 25/20 °C to 20/15 °C, that on the rain truck was somewhat lower, 20/17–18 °C. By comparison, the relative humidity rose as high as 98 % on the rain truck, falling back to ~80–84 % after 7 h.

**Table 1.** Experimental parameters

Experiment number	Start of drip and rain (day)	End of drip and rain (day)	Total duration of drip or rain (d)	Temperature shift^a^ (day)	Rain cycle^b^ Rain/pause (s)	Drip and rain water^c^ (amount)	Nutrients^d^ (amount)	First spike mature^e^ (day)
1	14	90	76	48	6/60	10 W	1N	75–83
2	14	87	73	59	1.8/85	2.5 W	1N	72–77
3	14	87	73	60	6/60	10 W	2N	77–85
4	45	87	42	59	12/55	10 W	1N or 2N	84–88

^a^Seeds were planted on day 1 and grown under constant 22 000 lux at 15/10 °C (16/8 h) until shifting to 23/18 °C (16/8 h) for seed maturation. The standard phytotron method (Dormling *et al.*, 1966) for delivering water by spraying at pot level (twice daily) and full-strength Hoagland nutrients (twice weekly) was used except in the time intervals when rain and drip are specified.

^b^From 0830 to 1230 h.

^c^10W and 2.5W correspond to 50 and 12.5 mm d^−1^, respectively, which are ~10 and 2.5 times the maximum amount of rain observed in Svaløf, Sweden, in the 1959–68 growing seasons. Number of litres of water passing through the rain and drip pots is given in Supplementary Data Table S1.

^d^Half-strength Hoagland (Went, 1957) was delivered every other day (1N) or every day (2N); for the standard phytotron method it was delivered at 0800 h and for drip and at 1500 h for rain.

^e^Additional details are given in Table 2. The average number of days to heading of the first spike ranged from 40 to 47.

**Table 2.** Days to maturity of first spike on ‘Bonus’, *cer.c36*, .*j59*,.*u42*, .*i16* and .e8 resulting from different watering^a^ and nutrient^b^ regimes (mean of 14 plants ± s.e.)

Water during vegetative phase					Water during maturation phase			
		Experiment 1	Experiment 2	Experiment 3			Experiment 4	
		10W, 1N	2.5W, 1N	10W, 2N			10W, 1N	10W, 2N
‘Bonus’	St p^c^	76.7 ± 0.7	76.1 ± 0.3	82.6 ± 0.5	‘Bonus’	Drip	84.1 ± 0.3	83.6 ± 0.4
	Drip	81.3 ± 1.0	72.4 ± 0.5	77.0 ± 0.6		Rain	85.1 ± 0.4	
	Rain	78.7 ± 0.7	74.6 ± 0.4	80.0 ± 0.8	*cer.u69*	Drip	85.0 ± 0.5	84.3 ± 0.3
*cer*.*c36*	St p	75.6 ± 0.5	76.0 ± 0.3	84.5 ± 0.4		Rain	87.4 ± 0.5	
	Drip	78.6 ± 0.7	73.6 ± 0.3	78.1 ± 0.6	*cer.c36*	Drip	84.9 ± 0.5	85.4 ± 0.3
	Rain	80.3 ± 1.0	74.2 ± 0.2	82.2 ± 0.7		Rain	88.0 ± 0.4	
*cer.j*59	St p	77.3 ± 0.3	74.9 ± 0.2	82.4 ± 0.4	*cer.i16*	Drip	85.4 ± 0.3	84.9 ± 0.4
	Drip	79.4 ± 1.0	76.7 ± 0.3	80.4 ± 0.5		Rain	87.2 ± 0.5	
	Rain	83.1 ± 0.6	76.4 ± 0.2	83.9 ± 0.4	*cer.e8*	Drip	84.7 ± 0.5	84.5 ± 0.4
						Rain	86.4 ± 0.3	

^a^10W and 2.5W, 19.7 and 4.9 L water d^−1^, respectively.

^b^1N, nutrients every other day; 2N, nutrients every day.

^c^Standard phytotron watering method.

At the time of the thermoperiodic shift, ‘Bonus’ had seven or eight heading spikes, meaning that heading begins during the vegetative phase (Dormling *et al*., 1966, 1969). In the first experiment the thermoperiodic shift took place earlier, at 48 d (Table 1). As this proved incompatible with many other phytotron experiments the shift thereafter took place at 59 or 60 d. In all experiments at least one spike per plant had headed by day 47. The number of ripe spikes in the present experiments ranged from 13.8 to 20.4 under standard phytotron watering conditions (Table 3), demonstrating that heading continues into maturation phase after starting in vegetative phase.

Giving nutrients every day (2N) during the vegetative phase led to a marked delay of ~5–9 d to maturity for all three genotypes when using the standard phytotron watering method, but not with the rain or drip methods (Table 2). Nutrient amounts likewise did not affect the day of maturity of the first spike (84–85) for all five genotypes (Table 2) when the drip method was used for delivering water during the maturation phase.

*Amount of water.* Maximum rainfall in a growing season of 5 months (April–August) from 1959 to 1969 at Svalöf, Sweden, was 407 mm (Swedish Meteorological and Hydrological Institute, Year Book 50 Part 2.1, Table 3). No excessive rainfall occurred during this time interval, in contrast to recent years. The equivalent amount of rain given in 76 d is 5.35 mm d^−1^, for example. As the circular area encompassing a 50 × 50 cm truck is 3935 cm^2^, 1.97 L d^−1^ is required, or, if the rain period is 4 h, 0.49 L h^−1^. Delivering ~2.5 and 10 times as much water (2.5W and 10W, respectively) requires 4.9 and 19.7 L d^−1^. To confirm that the planned relative water amounts were delivered every day, all the water running through the 14 pots on each rain truck was collected and measured (Supplementary Data Table S1).

The average (± s.e.) total number of litres collected for the 11 10W rain trucks was 762 ± 15 and for the three 2.5W rain trucks it was 191 ± 3, which gives the expected ratio of 4:1. In another experiment with water reduced to 1W for 73 d, the average (± s.e.) total number of litres collected was 102 ± 6 compared with 76 predicted from the 2.5W and 10W results (Supplementary Data Table S1). Variability and high standard error, however, combined with the difficulty in trying to maintain a uniform delivery to all 14 pots with only 0.49 L h^−1^ (cycle: 1 s rain followed by a 90-s pause) based solely on storage tank pressure to the drip trucks, demonstrated that a 1W experiment was not technically feasible, leading to its abortion in the late maturation phase. In both the 1W and 10W experiments, when the amount collected from drip trucks was compared with that collected on rain trucks, the former was less than expected: 57 and 85 %, respectively. Given that the surface area of the 14 pots on a truck was 30.8 % of the rainfall area, one expects to collect 0.61 L d^−1^ per truck. From the data one can calculate that considerably more was collected: in the 1W rain experiment an average of 1.39 L, in the 2.5W experiment 2.67 L and in the two 10W experiments 10.32 L. This implies that foliage was channelling rain into pots, partly explaining the collection of more water from a rain truck than the analogous drip truck.

**Fig. 3.**
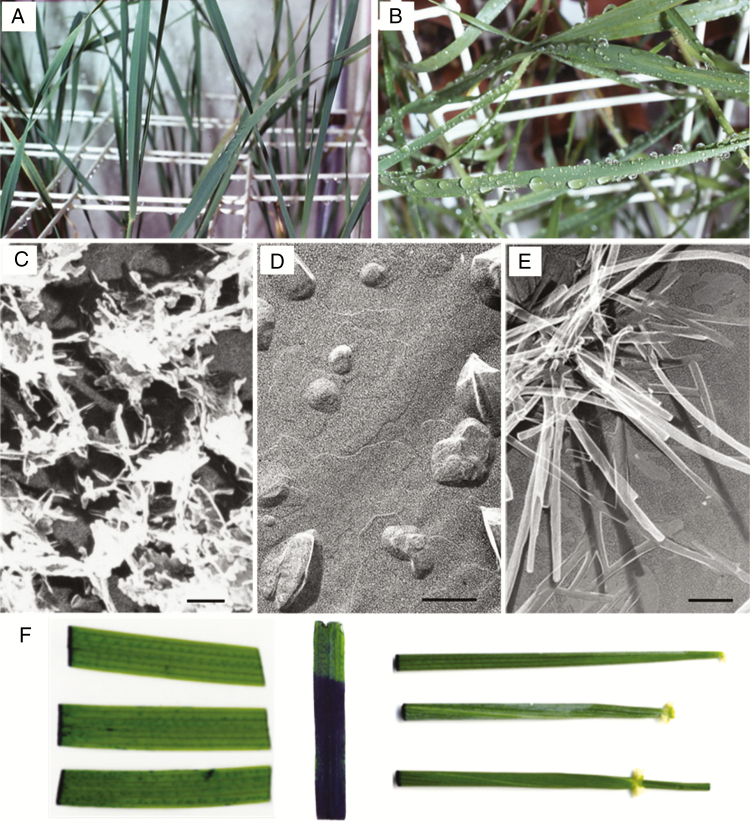
Leaf blades of wild-type ‘Bonus’ (A) do not retain water drops after rain, as do those of *cer.j59* (B). This results from the presence of many lobed plates on ‘Bonus’ (C) versus a few small mounds and closely appressed plates on *cer.j59* (D). Water sheds from vertical leaf sheaths, internodes and lemmas regardless of the presence of the long thin tubes characterizing ‘Bonus’. Tightly appressed to these surfaces are thin plates, which are visible on lemmas of *cer.i16*, which has fewer and shorter tubes (E). TB stain of leaf blades (F, left and centre) and sheaths (F, right): horizontal (top) ‘Bonus’; (centre) *cer.j59*; (bottom) *cer.c36*, vertical, *cer.zv268*. Scale bar = 1 µm.

*Wax phenotypes*. Rain drops do not readily run off barley leaf blade *eceriferum* mutants. Half an hour after raining on wild-type ‘Bonus’ only the divider bars retain some drops (Fig. 3A), whereas leaf blades of *cer.j*59 are still covered with drops (Fig. 3B). This phenomenon (Neinhuis and Barthlott, 1997) is caused by the many small lobed wax plates present on wild type (Fig. 3C) but absent on *cer.j*59, which has infrequent small wax mounds and thin plates closely appressed to the surface (Fig. 3D). While the uppermost leaf sheaths and exposed internodes plus spikes of ‘Bonus’ and *cer.j*59 are covered with long thin tubes, giving these organs a blue-grey colour, and genotypes that lack them, such as *cer.c*36 (von Wettstein-Knowles, 1972), are bright green (Fig. 1B, panel sides versus centre), the vertical nature of these organs results in water drop shedding.

In addition to ‘Bonus’ and *cer.c36*, three additional mutants having different spike wax coats were included in the maturation phase experiment: (1) *cer.u69*, which has highly lobed, large plates in addition to long thin tubes (von Wettstein-Knowles, 1972; Schneider *et al.*, 2016) on the uppermost leaf sheaths and exposed internodes plus spikes; (2) *cer.i16*, with a ~36 % reduction in the β-diketones in the total wax, resulting in shorter tubes peculiar to spikes (Fig. 3E); and (3) *cer.e8*, with a ~16 % reduction in β-diketones forming the long thin tubes only on spikes (von Wettstein-Knowles, 1972, 1976). The latter articles detail the methods used to grow ‘Bonus’, *cer.i16* and .e8, plus the details of the methods used to determine the composition of the acyl aliphatics in spike waxes. Given that both mutants are phenotypically glossy, major reductions in the total amount of waxes must accompany the 16 and 36 % reduction in DKS aliphatics (von Wettstein-Knowles 1976). A thin-layer chromatographic picture illustrating the reduction of the β-diketones in *cer.i16* spike waxes has been published (von Wettstein-Knowles, 1971).

*Electron microscopy.* The procedure for preparing leaf and lemma epidermal surfaces pre-shadowed with gold–palladium at an angle of 45° followed by deposition of 100–200 Å carbon while rotating at 75° under vacuum for examination in a Siemens Elmiskop 1A has been described (Netting and von Wettstein-Knowles, 1973).

*Traits measured.* For all 14 plants on each truck the following eight traits were measured: culm length of first tiller; spike length with awns on first tiller; number of ripe spikes at harvest; number of kernels on ripe spikes at harvest; air-dry weight of kernels on ripe spikes; days to heading of first spike; days to maturity of first spike; and straw dry weight. Additionally, 1000-kernel weight on ripe spikes was calculated and will be referred to as a trait here. The mean, standard deviation and standard error for each trait were calculated. For each experimental condition, *t*-tests were carried out to determine significance levels of mutant values versus those of the pertinent wild type, ‘Bonus’.

*Crude protein and dye-binding capacity.* The percentage of crude protein in the seeds was determined by measuring the N_2_ content of the seeds using the Kjeldahl technique and multiplying by 6.25 to convert N_2_ to protein. The dye-binding capacity (DBC) for basic amino acids in the crude protein extracts using Acilane Orange G was assayed as described (Munck *et al.*, 1970; Knoblauch *et al.*, 1977). The amino acid composition of seeds from ‘Bonus’, *cer.c36* and .*j59* was determined as described previously (Munck *et al.*, 1971). Pearson’s analysis was carried out to determine the strength of the correlation between percentage protein content and DBC.

### Water loss experiments.

Seeds of *cer.xa838*, *.ya180*, *.yg1014*, *.yl187*, *.yl188*, *.ym130*, *.ym753*, *yo647*, .*yp949*, *.yq1246*, *.yu158*, *.zk85*, *.zq214*, *.zv268*, *.zv342*, *.zy118* and *.zz615*, plus *wlt2*, *wlt15* and *wlt16*, were obtained from the Nordic Genetic Resource Center, Alnarp, Sweden (www.nordgen.org). Seeds of all other mutants and cultivars were sourced in-house. They were planted and grown in a non-environmentally controlled greenhouse at Lund University until the appropriate leaves were fully expanded.

Three centimetres of ~5-cm central segments of leaf blades and sheaths were immersed in 0.05 % (wt/vol) toluidine blue (TB) for 3 h and then washed under water to remove non-bound dye (Richardson *et al.*, 2007) before scanning. To ascertain non-stomatal water loss, ~10-cm-long central segments of fully expanded leaves were placed across pre-weighed polystyrene weighing boats and weighed every hour for the first 4 h and then at 9 or 10, 24, 48, 72, 96 and 120 h. Thereafter most leaves were vacuum-freeze-dried overnight to obtain dry weights.

## RESULTS

### Barley *Cer* genes: water loss and rain

Both cutin and intracuticular waxes contribute to the barley cuticle’s water retention (Li *et al.*, 2013; Richardson *et al.*, 2007). To ascertain whether these apoplastic components underlying the epicuticular waxes are markedly affected in barley *cer* leaf mutants, leaf blade and sheath segments were immersed in TB. Tissue from ‘Bonus’, *cer.c36* and *.j*59 was not stained (Fig. 3F), indicating that the examined tissues of these two mutants were not defective with respect to water loss. By comparison, leaves from *cer.zv268* and .*ym130* mutants, as illustrated in Fig. 3F and Supplementary Data Fig. S1, respectively, stained strongly as reported (Li *et al*., 2013, 2015). Analogous results (not shown) were obtained for *cer.zv342* and .*ym753*. When leaves of representative mutants of the other 24 *Cer* genes with only a modified leaf *cer* phenotype (Lundqvist and Lundqvist, 1988) were exposed to TB, however, they remained unstained, as did *cer.zk85* and .*yg1014*, appearing to lack wax on all organs (Supplementary Data Fig. S1). Likewise, two *cer.yl* mutants obtained from the Nordic Genetic Resource Center, belonging to the uppermost internode plus leaf sheath and spike *cer* group, did not stain with TB (Supplementary Data Fig. S1) as expected, in contrast to results reported by Li *et al.* (2017), working with the Bowman-derivative, near-isogenic line BW143.

**Fig. 4.**
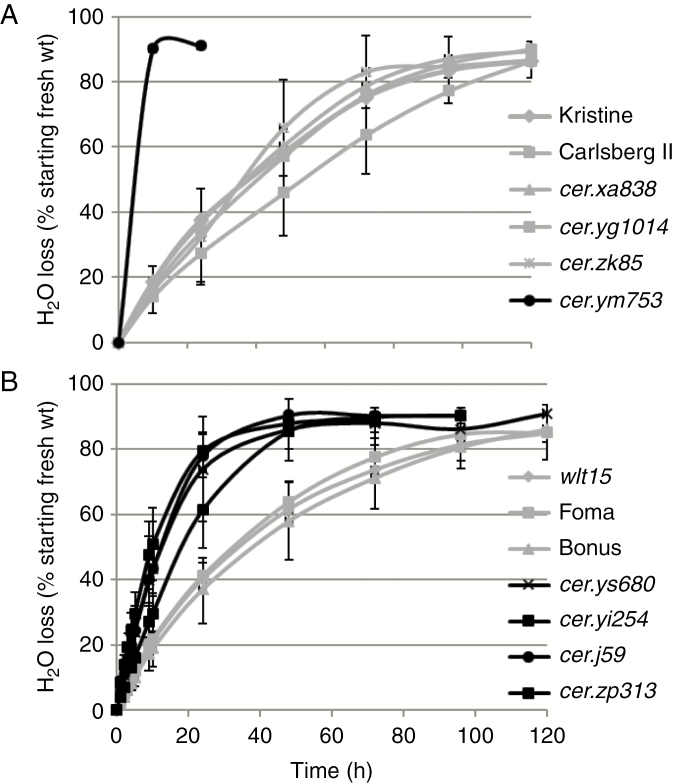
Based on the rate of non-stomatal water loss, four barley cultivars and the *cer* mutants can be divided into three groups. In (A), *cer.ym753* (group 1, black line) loses its water within 4 h while the two cultivars and three other *cer* mutants (group 3, grey lines) take 72–96 h. In (B) the two cultivars plus *wlt15* (group 3, grey lines) take 72–96 h to lose the same amount of water as the four *cer* mutants in group 2 (black lines) do in 24–48 h. *n* ≥ 6.

An early study of 14 *cer* leaf barley mutants looked for a possible influence of wax structure on non-stomatal water loss at 6 h (Larsson and Svenningsson, 1986). No effect was found. In the present study of all barley leaf *Cer* genes plus three *wlt* mutants, the rate of non-stomatal water loss was followed until ~10 % of the starting fresh weight remained (Fig. 4; Supplementary Data Fig. S2). Stomatal water loss was assumed to be unchanged since in a screen of epidermal images from the *cer* leaf and *wlt* mutants none was identified with a modified structure or frequency of stomates (Bonnell, 2012). No differences were discerned in the phenotypes exhibited by the leaves of the investigated genotypes during water loss. Three groups were disclosed. Group 1, comprising *cer.zv* and *.ym* mutants, lost water very quickly, as illustrated by *cer.ym753* (Fig. 4A), confirming published results (Li *et al*., 2013, 2015). Group 2, consisting of *cer.j*, *.yi*, .*ys* and .*zp* mutants, lost water faster than the other *cer* leaf mutants and their respective wild-types in group 3 (Fig. 4B), but nowhere near as rapidly as group 1 mutants. The three tested *wlt* mutants belonged to group 3, as did *cer.zk85* and *.yg1014*, appearing to lack wax on all organs (Supplementary Data Figs S1 and S2). If the rate of loss were linear, groups 1, 2 and 3 would lose 22.9, 1.7 and 1.25 %, respectively, of starting water per hour based on data in Fig. 4. According to which time point is used, the apparently small difference between groups 2 and 3 is significant (*P* < 0.01 or *P* < 0.001).

### Identification of additional phenotypic traits associated with *cer.c36* and *.j59* mutations

**Fig. 5.**
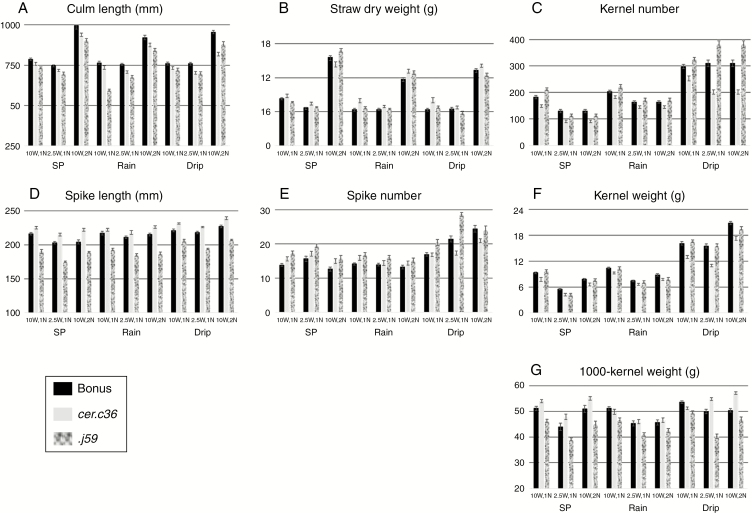
Effects of nine different environments during the vegetative phase on seven phenotypic traits (A–G) of ‘Bonus’, *cer.c36* and *.j59*. Variation in the environments was achieved using three watering regimes [standard phytotron (SP), rain and drip] with two amounts of water (10W and 2.5W, 19.7 and 4.9 L water d^−1^, respectively) plus nutrients every other day (1N) or every day (2N). Average of 14 plants ± s.e.

Before investigating whether the watering regimes affected vegetative growth and yield, phenotypes of *cer.c36* and .*j*59 were characterized using different watering methods and nutrient amounts and time of the shift to maturation conditions. Observed trait phenotypes under nine environmental regimes are presented in Fig. 5 and Supplementary Data Tables S2–S4. Supplementary Data Table S2 contains results from the standard phytotron watering method and Supplementary Data Table S3 those from the rain method. Anticipating that nutrients could prove limiting on rain trucks because of the increased volume of water running through pots compared with standard phytotron watered trucks, drip trucks were included in each experiment, the results of which are presented in Supplementary Data Table S4. The tables include significance levels of the differences between each mutant and the wild-type ‘Bonus’.

Figure 1A combined with the data in Fig. 5A, D, reveals that *cer.j59* was significantly shorter than ‘Bonus’ in all nine environments. While the length of culms contributed to this, the spikes were dramatically shorter. The culm lengths of *cer.c36* were always less than those of ‘Bonus’, but only in six of nine environments was the difference significant. Unexpectedly, *cer.c36* spikes were always longer than those of ‘Bonus’, being significantly so in seven of nine environments. No consistent differences between the mutants’ straw dry weights and spike numbers (Fig. 5B, C) occurred. The lack of concordance implies that these changes cannot be ascribed to either the *cer.j59* or the *.c36* mutation. Both the number of kernels and their weight (Fig. 5C, F, G) from *cer.c36* plants were always significantly less than those from ‘Bonus’. By comparison, no significant concordant differences between *cer.j59* and ‘Bonus’ were noted for these traits (Fig. 5C, F). Despite this, when the 1000-kernel weights were calculated for *cer.j59* they were always significantly less than those for the pertinent ‘Bonus’ control [‘Bonus’, 49.1 ± 1.03 (s.e.); *cer-j59*, 44.1 ± 1.12; *P* < 0.01] (Fig. 5G; Supplementary Data Tables S3 and S4). The drip watering regime led to an increase in spike number of all three genotypes that likely contributed to an increased number of heavier kernels (Fig. 5C, E, F).

Based on the above observations, alteration of the wax coats on *cer.j59* and .*c36* mutants was accompanied by other phenotypic changes independent of the thermoperiod shift time, amount and type of watering regime and amount of nutrients: *cer.j59* plants were characterized by shorter culms and spikes plus lesser 1000-kernel weights, while *cer.c36* plants had fewer and smaller kernels.

### Phenotypes of ‘Bonus’ traits versus those of *cer.c36*, *.u69*, *.i16* and *.e8*

To extend the above observations from experiments 1–3 with differential watering throughout the vegetative phase, an additional experiment (experiment 4) was carried out in which 10W was delivered only during the maturation phase (Table 1). This amounted to 77 % more water per day than in experiments 1–3, where the same amount was distributed over 73–76 d (Supplementary Data Table S1). Beyond ‘Bonus’ and *cer.c36*, three additional mutants having structurally different wax coats on their spikes were included: *cer.u69*, *.i16* and *.e8* (von Wettstein-Knowles, 1972, 1976). Compositional analyses of the KCS-derived aliphatics in *cer.i16* and .*e8* spike waxes revealed that the only significant change compared with ‘Bonus’ was a marked reduction in *cer.e8* of the C_31_ alkane, which was compensated for by increases in the C_23_, C_25_ and C_27_ alkanes (Supplementary Data Table S5).

**Fig. 6.**
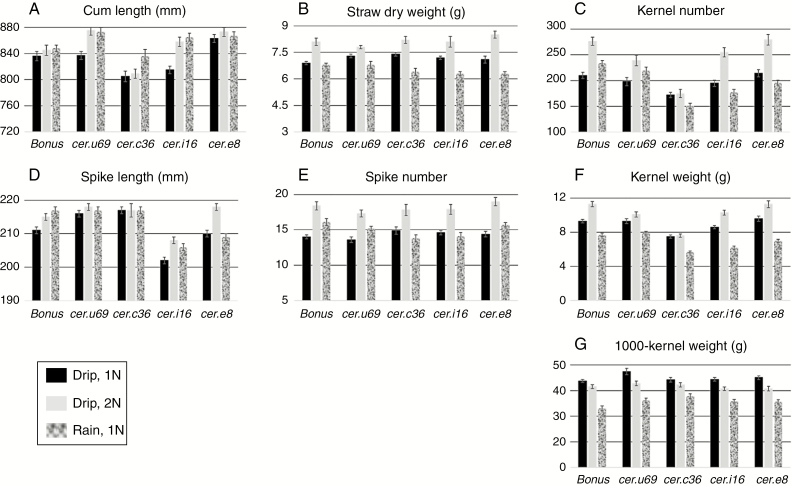
Effects of three different environments during the maturation phase on seven phenotypic traits (A–G) of ‘Bonus’, *cer.u69*, *.c36*, *.i16* and *.e8*. Variation in the environments was achieved using two watering regimes (rain and drip) with 10W (19.7 L water d^−1^) plus nutrients every other day (1N) or every day (2N). Average of 14 plants ± s.e.

Figure 6 presents the results of delivering 10W to drip and rain trucks only during the maturation phase. As in the vegetative phase experiments (1–3) *cer.c36* kernel numbers and weights were significantly less than those of ‘Bonus’ kernels (Fig. 6C, F; Supplementary Data Table S6) under the three environmental conditions tested. Rain and drip watering only during maturation consistently resulted in several other mutation-specific changes, namely, for *cer.e8* plants significantly longer culms but not spikes, and for *cer.i16* plants significantly shorter spikes but not culms compared with those on ‘Bonus’ plants (Fig. 6A, D). No additional phenotypic changes were observed for *cer.u69* mutants.

### Consequences of differential vegetative phase watering for ‘Bonus’, *cer.j59* and *.c36* phenotypes

**Fig. 7.**
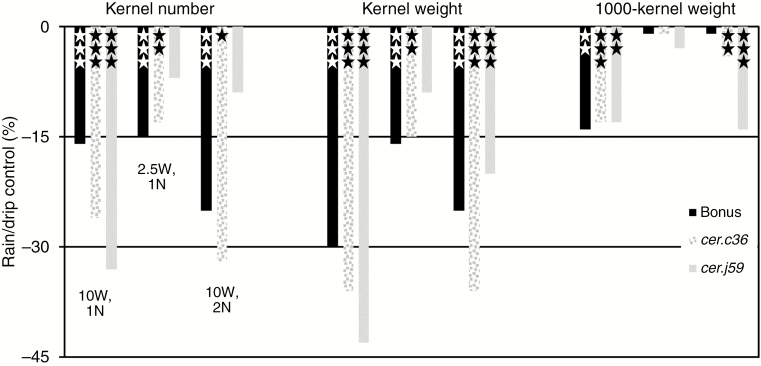
Rain throughout the vegetative phase alters yield phenotypes. Leftmost bar in each group is ‘Bonus’ with crystals on all organs, centre is *cer.c36* lacking crystals on uppermost internodes and leaf sheaths plus lemmas, and rightmost is *cer.j59* lacking crystals on leaf blades. For each panel from left to right the first group of three bars had 19.7 L water d^−1^ and nutrients every other day (10W, 1N), the second had 4.9 L water d^−1^ and nutrients every other day (2.5W, 1N) and the third had 19.7 L water d^−1^ and nutrients every day (10W, 2N). *P < 0.05, **P < 0.01, ***P < 0.001.

A comparison of rain versus drip watering outcomes in the vegetative phase in ‘Bonus’, *cer.j59* and *.c36* can be obtained by comparing the pertinent columns in Fig. 5 and values in Supplementary Data Tables S3 and S4. Three traits, namely kernel weight and number plus 1000-kernel weight, all decreased (Fig. 7), whereas culm and spike length plus straw dry weight and spike number were either unaffected or showed random effects (Supplementary Data Fig. S3). Specifically the yield traits (kernel weights and numbers) were consistently less in the rain by as much as 42 %. Only in two of 18 comparisons with kernel number and weights (both with *cer.j5*9) were differences insignificant, implying that wax structure changes indeed affected yield in the present experimental conditions. The decreases in 1000-kernel weights were never greater than 14 %. For all three yield traits the effect was least in the 2.5W experiments (Fig. 7, middle group of bars in each panel). These results demonstrate that neither the densely lobed plates characteristic of wild-type barley leaves from which rain readily sheds (Fig. 3A) nor the few mounds of wax with closely appressed thin plates which retain water drops on *cer.j59* leaves (Fig. 3B) are effective raincoats. That raincoat structures are important, however, is implied by the differences in the extent of the decrease resulting from rain. Given vegetative phase watering, whether or not the dense coating of long, thin tubes on the uppermost leaf sheaths and exposed internodes plus spikes of ‘Bonus’ barley protect the plant better than an almost smooth layer of wax does on these organs, as characteristic for *cer.36*, is indeterminable from these results.

### Consequences of differential maturation phase watering for ‘Bonus’, *cer.c36*, *.u69*, *.i16* and *.e8* phenotypes

**Fig. 8.**
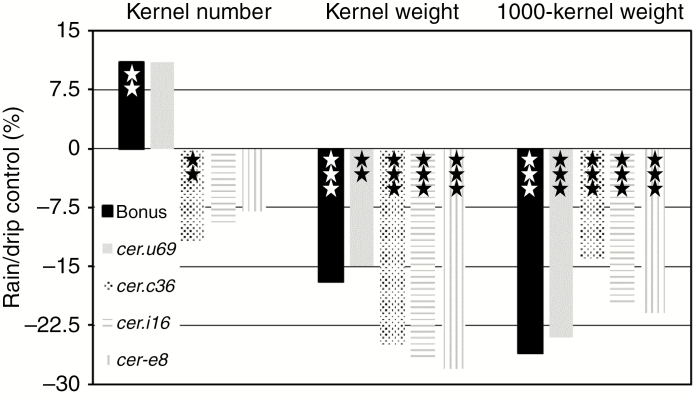
Rain in the maturation phase alters phenotype. From left to right in each panel are shown ‘Bonus’ with crystals on all organs, *cer*.*u69* with modified crystals on uppermost internodes and leaf sheaths plus lemmas, .*c36* lacking crystals on uppermost internodes and leaf sheaths plus lemmas plus .*i16* and .*e8* with reductions of crystals on lemmas. All plants received 19.7 L water d^−1^ and nutrients every other day (10W, 1N). **P < 0.01, ***P < 0.001.

Results of rain versus drip watering during the maturation phase were significant decreases from 7.5–9.3 to 5.7–7.9 g in kernel weight and from 44.3–45.1 to 33.1–37.9 g in 1000-kernel weight in all five genotypes (Fig. 8; Supplementary Data Table S6). Interestingly, although rain stimulated kernel number in ‘Bonus’ and *cer.u69* plants, rain suppressed kernel number in the other three mutants (Fig. 8). The extent and direction of the changes resulting from rain during the maturation phase on the three ‘Bonus’ and *cer.u69* yield phenotypes were analogous and contrasted with the other three mutants, characterized by decreases in all three yield phenotypes (Fig. 8). Also for the other four traits (culm length, spike length, straw dry weight and number of ripe spikes) *cer.u69* exhibited changes concordant with ‘Bonus’, although of different magnitudes (Supplementary Data Fig. S4). Supplementary Data Fig. S4 also reveals that straw dry weight for all four mutants was significantly less with rain during the maturation phase. Combined, these results imply that the wax coat structure on the uppermost leaf sheaths and internodes plus spikes influences yield.

Doubling the nutrients (2N) with drip watering during the maturation phase resulted in increases in straw dry weight, spike number, kernel number and kernel weight (Fig. 6). That all increases were significant except for *cer.c36* kernel number and weight indicates that this is another pleiotropic effect of this mutation. By comparison, giving 2N with all three watering regimes during the vegetative phase resulted in consistent, significant straw dry weight and culm length increases (Fig. 5).

‘Bonus’ and *cer.c36* were included in both the vegetative and maturation phase experiments. Supplementary Data Table S7 compares the results for four traits (culm length, kernel weight, kernel number and 1000-kernel weight) with drip and rain watering in the two phases (1N with rain and drip watering in Table S7A; 2N with drip watering in Table S7B). With one exception all comparisons in Supplementary Data Table S7B reveal significant differences, in which the result in the vegetative phase is greater than that in the maturation phase. Comparisons in Supplementary Data Table S7A reveal that culm length, kernel weight and kernel number were less with the vegetative than the maturation phase watering regime, while 1000-kernel weights were significantly greater. Thus, average 1000-kernel weight was 40.5 ± 1.6 g (s.e.) for those with the maturation phase versus an average of 51.3 ± 1.6 g for those with the vegetative phase watering regime.

### Crude protein content and DBC of ‘Bonus’, *cer.c36* and *.j59* kernels

**Fig. 9.**
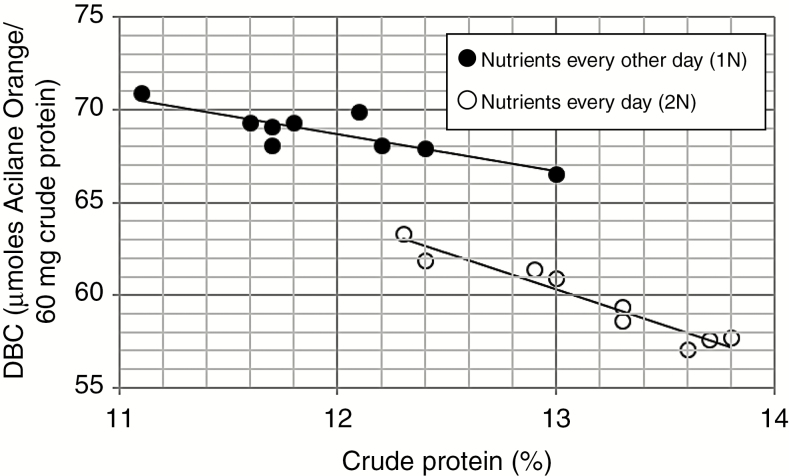
Correlation between crude protein content and DBC of kernels of ‘Bonus’, *cer.j59* and *.c36* from the different watering regimes of experiments 1 and 3 depends on the nutrition level.

To test whether watering method and nutrient amount affected protein content and/or the percentage of basic amino acids in kernels, crude protein was determined as well as the DBC. Kernels from two plants of each of three genotypes subjected to three different watering regimes and two different nutrient amounts in experiments 1 and 3 were analysed. Despite the small sample size, suggestive differences were revealed. Crude protein from the three genotypes having indistinguishable amino acid compositions varied from 11.1 to 13.8 % while DBC ranged from 57.1 to 70.9 µmoles Acilane Orange G bound to 60 mg crude protein (Supplementary Data Table S8). In phytotron experiments determining optimum conditions for maturation of ‘Bonus’ barley that are most similar to those employed here, a protein content ranging from 13.1 to 15.0 % was negatively correlated to DBC from 62.4 to 64.2 (Dormling *et al.*, 1969). An analogous negative correlation was found for all data herein, with *R*^2^ = 0.834. Results cannot be subdivided according to watering method or genotype, but can be on the basis of nutrient amount, as shown in Fig. 9, which is based on the data in Supplementary Data Table S8. With 1N a lower range of crude protein percentage was correlated with a higher range of DBC, while with 2N the opposite occurred, the former’s slope being less (−0.237) than the latter’s (−0.359). In all comparisons 2N DBC was less than the analogous 1N value, while with one exception the crude protein percentage was higher (Supplementary Data Table S8). The present results extend the earlier study with Hoagland nutrients given once a week combined with half the normal amount of water by the standard phytotron method versus twice a week with the normal amount of water. With one exception the former resulted in lower percentages of crude protein, and even though the overlap between the two groups was considerable (Dormling *et al.*, 1969) a similar relationship between nutrition and crude protein percentage was deduced here.

## DISCUSSION

Plant cuticles must inhibit not only water loss but also nutrient loss. Both these functions have been probed here. (1) The contributions of wax structure and humidity were revealed under simulated rain in varying amounts. Highly significant reductions in kernel yield resulted from the increase in humidity regardless of the genotype and its wax structures. The latter, however, conceivably contributes given the different extents of the decreases. (2) Major defects in the cuticle structure of 28 *Cer* leaf genes and three *wlt* mutants were screened for using TB staining, and the rate of non-stomatal water loss was determined. None of the genotypes, except *cer.zv* and .*ym*, played significant roles. (3) Measuring other phenotypic traits of five *Cer* genes in a range of environmental conditions uncovered new effects as well as confirming known pleiotropic effects.

### Rain, humidity, wax structure and yield

To investigate whether morphological changes in epicuticular wax influence kernel yield in the presence of excess moisture, rain was distributed as a fine spray throughout the vegetative stage for 4 h every day. This increased the relative humidity from ~83 to 98 %, which then decreased over the next 8 h to 83 %. During this time the temperature was also 2 °C lower on rain trucks. When 2.5W was given, kernel weight compared with the drip control decreased for ‘Bonus’, *cer.j59* and *.c36* (by 16, 9 and 15 %, respectively). Likewise, kernel numbers were reduced. Yield differences under the specified rain parameters thus occurred irrespective of whether leaves were decorated with a dense coat of lobed plates and uppermost leaf sheaths, exposed internodes and spikes with long thin tubes or by thin, flat plates closely appressed to either cuticle surface. This suggests that epicuticular wax structure has little effect on kernel yield under these conditions. Increasing the rain amount 4-fold (10W), however, resulted in highly significant, additional decreases in kernel weights of 33 % for *cer.j59* and 27 % for *cer.c36*, but only an additional 13 % for ‘Bonus’. Significantly fewer kernels were present on all three genotypes after the greater amount of rain. While in some comparisons of kernel number and weight increasing the nutrient amount reduced the yield decrease resulting from greater rain amounts, in no case was it prevented. These results imply that if plants are exposed to enough rain the wax structure can influence kernel yield.

Decreased yields resulting from 10W rain in the vegetative versus maturation phase are not directly comparable because: (1) almost twice as much rain was delivered per day in maturation than vegetative phase experiments, and (2) leaves and spikes contribute differently to kernel filling. Given the similar wax load and dominating wax structure of tubes on *cer.u69* and ‘Bonus’ (von Wettstein-Knowles, 1972), the analogous effect of rain on kernel number and weight is unsurprising. The similar greater kernel weight reductions after exposure to rain for *cer.c36*, *.i16* and *.e8* versus ‘Bonus’ and *cer.u69* plants cannot be correlated with the striking structural modifications of their wax coats. The results obtained for the maturation phase, however, are in accord with those from the vegetative phase experiments. Namely, given enough rain kernel yield is lower in the specified mutants than in the wild type, implying a minor role for epicuticular wax in leaching.

Metabolite leaching through leaf cuticles was conclusively demonstrated many years ago using radioisotopes (Long *et al.*, 1956). Carbohydrate was the major leachate component (Tukey, 1970). The leaching agent was water in the form of dew, mist, fog or rain, a light drizzle being more effective than a downpour. Moreover, if leaching continued for several days, metabolite translocation from other plant parts was required for their replacement. The marked humidity effect on transport of ionic compounds through deduced polar pores to the cuticle’s inner side has been demonstrated. Sugars, amino acids and ions are presumed to diffuse through the same pores to reach the cuticle surface (Schreiber, 2005). More recently the word ‘pore’, implying a more or less permanent passage through the cuticle, has been replaced with the phrase ‘dynamic aqueous continuum’, reflecting a cuticular network connecting random hydrophilic domains that are highly dependent upon hydration level (Fernández *et al.*, 2017). The present results with very high humidity resulting from raining strongly support the idea that this mechanism for transport of water and electrolytes across cuticle surfaces for alpine conifers and tree-line species (Fernández *et al.*, 2017) also applies to the herbaceous species barley.

Leaching has been extensively exploited in recent years to characterize microbial phyllosphere inhabitants (Leveau and Lindow, 2001; Vorholt, 2012) and has led to a model of sugar diffusion across the cuticular layer of the apoplast (van der Wal and Leveau, 2011). In the present experiments, insufficient translocation of substrates, water soluble carbohydrates and nitrogen mobilized from proteins via the phloem (Schnyder, 1993; Gebbing and Schnyder, 1999) under intense rain resulted in significant decreases in kernel weight. The final destination of substrates among major components of barley kernels, ~78–85 % carbohydrate, 8–11 % protein and 2–3 % lipids (Briggs, 1981), however, was apparently unchanged since the kernels’ protein percentage and its amino acid composition were unaffected by rain in the wild type, *cer.j59* and *.c36*.

### Water deficit and barley leaf *Cer* genes

Water deficit has been the driving factor for studying the relationship between glaucousness and agronomic performance in the Gramineae. To ascertain whether barley leaf *cer* mutants had altered cuticle structure and as a result were more susceptible to water loss than their mother cultivars, TB staining was carried out. Except for *cer.zv* and .*ym* mutants determining a GDSL-motif esterase/acyltransferase/lipase gene (Li *et al.*, 2017), none of the other 29 tested *cer* mutants and three *wlt* mutants were stained by TB. Although accounting for only a small percentage of water loss, the predominant epidermal pavement cells have the same type of wax crystals in analogous amounts to the stomates. Thus, non-stomatal water loss was ascertained to reveal whether wax structure participates in this phenomenon. The results revealed that *cer.ys*, *.yi*, *.j* and *.zp* lose water at a significantly slightly faster rate than the four cultivars and other mutants. For example, they lost 80 % of their starting water in 48 h, a period during which the cultivars and other mutants lost 60 %. The *cer.zv* and .*ym* mutants lose essentially all their water in 3.5 h. Given that water loss is linear, the *cer.zv* and *.ym* mutants lose water 13.5 times faster than *cer.ys*, .*yi*, *.j* and *.zp* and 18.3 times faster than the four cultivars and other mutants. These data lead to the conclusion that, with the exception of *cer.zv* and .*ym*, none of the other barley leaf *Cer* genes or the three *wlt* mutants play very significant roles, if any, in preventing water loss.

### Associated pleiotropic effects of the studied *cer* mutants

The present results extend previous findings and reveal new phenotypic modifications caused by mutations in the *Cer.i*, *.e*, *.j* and *.c* genes. None was associated with *cer.u69.* This indicates that the *Cer.u*-encoded hydroxylase (Schneider *et al.*, 2016) may well be limited to hydroxyl insertion into the β-diketone carbon chain, the final biosynthetic step in the DKS pathway. The reduction in DKS-derived aliphatics resulting in fewer of the long thin tubes in the *cer.e8* spike waxes was accompanied by a significant reduction in the C_31_ alkanes as well as a significant increase in culm length compared with that of ‘Bonus’. By comparison, the reduction in DKS-derived aliphatics characterizing *cer.i16* resulting in fewer and shorter wax tubes was accompanied by a significant decrease in spike length, but not by a compositional change in the aliphatic lipids versus those of ‘Bonus’. The surprising diversity in phenotypes that are affected by a single *cer* mutation is hardly surprising given that the wax components not only have to be synthesized but also have to be transported to the surface, with both processes requiring high levels of regulation and coordination. For example, expression studies of *OsABCG9* imply that its product transports not only epidermal wax aliphatics but also other lipid-like molecules in different types of cells (Nguyen *et al.*, 2018).

A consonant pleiotropic effect of *cer.j59* in all experiments reported here, beyond its marked effect on wax composition and load (Giese, 1976), is a height reduction of ~10 % for both culms and spikes, analogous to 8 % in the field (Baenziger *et al.*, 1983). Likewise, with respect to 1000-kernel weights, previous field results (Baenziger *et al.*, 1983) and those reported here were 9–14 % less for *cer*.*j* mutants than their mother cultivars. While field kernel weights of *cer.j59* were reduced (Baenziger *et al.*, 1983), in the present and earlier phytotron experiments those of the mutants were elevated by 3–4 % (Gustafsson, 1963). Thus, according to which trait is used to compare yield, essentially no effect or a reduction of ≥9 % can be attributed to *cer.j* non-glaucosity.

Previous field and phytotron experiments with *cer.c3* and .*c36* resulted in kernel yields 6–18 % less than those of ‘Bonus’ (Gustafsson, 1963; Gustafsson *et al.*, 1975), which is similar to the 15 % less for *cer.c36* obtained here using standard phytotron watering conditions with various nutrient amounts. An isoline of barley cultivar ‘Troubadour’, having only a trace of a bloom on the uppermost leaf sheaths and spikes (Febrero and Araus, 1994), however, did less well, with a yield ~73 % of that of the glaucous mother cultivar in 2 years in the field at Barcelona University (Febrero *et al.*, 1998). Combining all rain and drip experiments reported here reveals that *cer.c36* kernels weighed 11–35 % less than those of ‘Bonus’. A second concordant pleiotropic effect of *cer.c36* in the present and original phytotron experiments is a reduction in kernel number ranging from 11 to 37 %. Although cognate ranges in kernel number and weight reduction were found they were uncorrelated, so that 1000-kernel weight was inconsistently affected. An interesting question for the future is how mutation of *Cer.C* encoding the polyketide synthase DKS (Hen Avivi *et al*., 2016; Schneider *et al.*, 2016) results in these pleiotropic effects.

### Conclusions

Plant cuticles must protect not only against water loss but also nutrient loss via leaching. Here both functions were investigated in barley. Mutants of 28 *Cer* leaf genes and three *wlt* mutants were screened for rate of non-stomatal water loss and reaction to the TB stain. With the exception of *Cer.zv* and .*ym* mutations, none of those studied in the *Cer* genes or *wlt* mutants significantly affect water retention. Epicuticular wax structure determines how long water drops persist on the cuticular apoplast surface in contact with the phyllosphere. For a long time humidity in the phyllosphere has been known to influence nutrient leaching. Recently this has been envisaged to occur via a cuticular network of hydrophilic domains rather than pores (Fernandez *et al.*, 2017). Does epicuticular wax structure play a role in this process? To answer this question the barley cultivar ‘Bonus’ and *cer* mutants were grown under three to 12 different environments in a phytotron and subjected to simulated rain, effecting a humidity increase, during the vegetative or maturation growth phases. A highly significant extent of leaching was deduced from the decreases in kernel yield, implying significant nutrient loss regardless of wax structure. Given the different extents of the decreases, however, wax structure conceivably contributes. The experimental setup employed will be useful for investigating the mechanism of leaching in herbaceous species.

Measuring other phenotypic traits uncovered new effects and confirmed and extended to a wider range of environments previously noted pleiotropic effects. Interesting questions for the future are the bases for the pleiotropic effects of the *Cer.i*, *.e, .j* and *.c* mutations. For example, how does a mutation in *Cer.c*, encoding the polyketide synthase DKS essential for synthesis of the β-diketone aliphatics (Hen-Avivi *et al.*, 2016; Schneider *et al.*, 2016), also result in decreased kernel size and number? Why does mutation of *Cer.j* have such a marked effect on wax composition and load (Giese, 1976) while simultaneously reducing plant height and 1000-kernel weight? Where in metabolism do the genes function that both influence the DKS pathway and determine either shorter spikes in *Cer-e* mutants or longer culms in *Cer-i* mutants?

## SUPPLEMENTARY DATA

Supplementary data are available online at https://academic.oup.com/aob and consist of the following. Figure S1: results of toluidine blue staining. Figure S2: water loss of barley cultivars and mutants. Figure S3: effect of simulated rain in the vegetative phase on growth traits. Figure S4: effect of simulated rain in the maturation phase on growth traits. Table S1: litres of water running through pots subjected to the drip and rain watering regimes. Table S2: vegetative and yield traits resulting from the standard phytotron watering regime in the vegetative phase. Table S3: vegetative and yield traits resulting from the rain watering regime in the vegetative phase. Table S4: vegetative and yield traits resulting from the drip watering regime in the vegetative phase. Table S5: composition of five wax classes from spikes of ‘Bonus’ and its mutants *cer.i16* and .*e8*. Table S6: vegetative and yield traits resulting from the drip and rain regimes in the maturation phase. Table S7: comparison of vegetative and maturation phase data for four ‘Bonus’ and *cer.c36* phenotypic traits. Table S8: crude protein and dye-binding capacity of kernels from ‘Bonus’, *cer.c36* and *cer.j59* plants grown under different regimes of 10W and nutrients throughout the entire vegetative phase of growth.

mcaa086_suppl_Supplementary_Material
