# Supplementary material for: Ecophysiology with barley eceriferum (cer) mutants: the effects of humidity and wax crystal structure on yield and vegetative parameters
Source: Ann Bot. 2020 May 3;126(2):301–13. doi: 10.1093/aob/mcaa086 (PMC7380459; doi:10.1093/aob/mcaa086)
Supplement: mcaa086_suppl_Supplementary_Material [file mcaa086_suppl_supplementary_material.docx]

Fig. S1. Results of three hour toluidine blue staining of segments of leaves from two barley cultivars, 29 *cer* leaf mutants, 2 *cer-yl* mutants with wild type leaves plus two *wlt* mutants. Leaf segments are from the second leaf except for the three mutants, *cer*.*zy118*, .*zq248* and .*zg214*, in which the flag leaf was used as only the wax on the upper three leaves has a *cer* phenotype.

*.zk85*

*.ya180*

*.yj667*

*.yq1246*

*.yf652*

*.yo647*

*.yb200*

*.xa838*

2nd


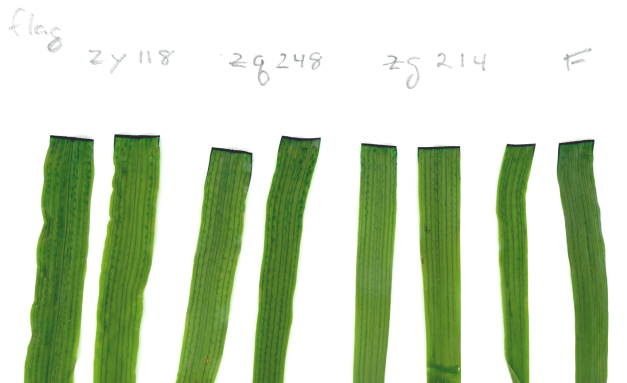

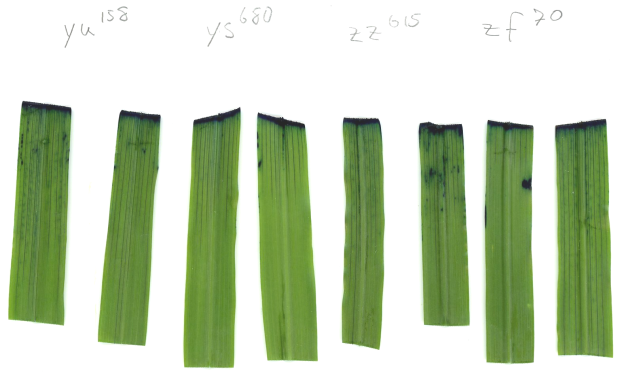


*.yu158*

*.ys680*

*Foma*

*.zg214*

*.zq248*

*.zf70*

*.zy118*

*.zz615*

2nd

flag

2nd

Bonus

*.zp313*

*.yi254*

*.ye267*

*.ze81*

*.za227*

*.zd67*

*.zj78*

*.p37*

*.j62*

*.j188*

*.yp949*

*.zh54*

Bonus

.*yl188*

*.yl187*

*.ym130*

*wlt 2*

*wlt 15*

2nd

*.yg1014*


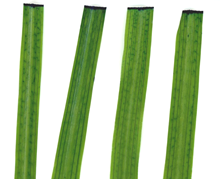


Fig. S2. Non stomatal water loss ± SD after 24 hours at room temperature and circa 40% relative humidity as % starting water (a-d) or % starting fresh weight (e). In a-d, n is ≥ 6; in e, n ≥ 5 except for *cer.yu*, n = 1, *cer*.*yq,* n=3, *cer*.*xa* and *cer*.*yp*, n=3. Results analogous to those for *cer-ym753* were obtained for *cer.zv.286* and .*342.*


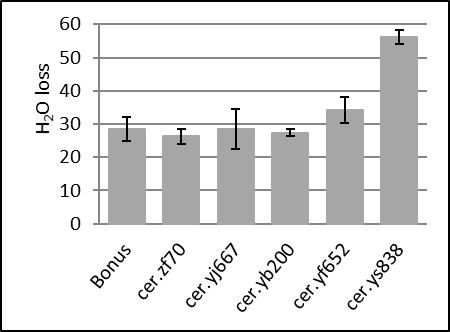

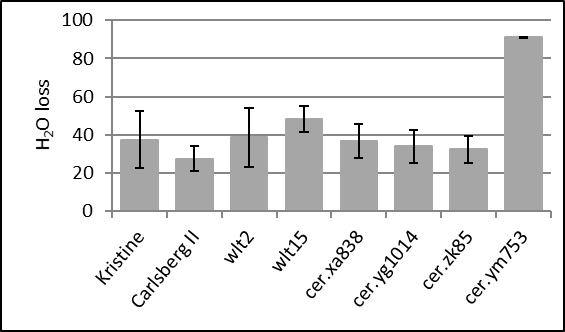


a

b


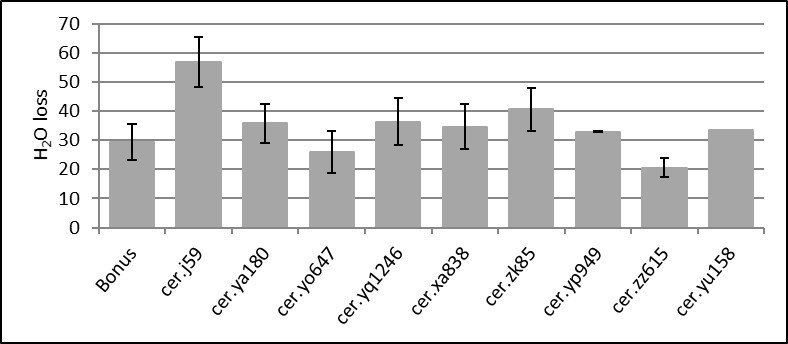


e


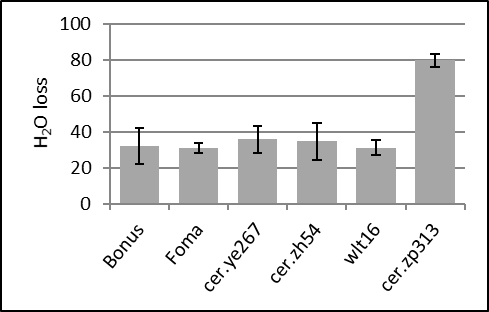


c


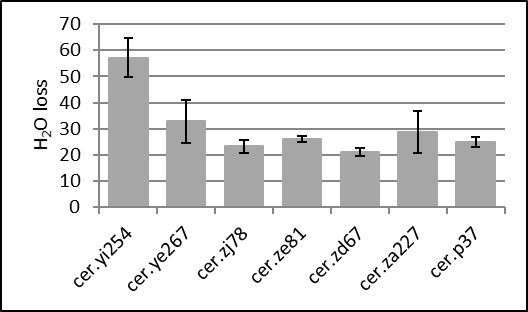


d

Fig. S3. Effect of rain in the vegetative phase on growth traits. Leftmost bar in each group is Bonus with crystals on all organs, center is *cer.c36* lacking crystals on uppermost internodes and leaf sheaths plus lemmas and rightmost is *cer.j59* lacking crystals on leaf blades. For each panel from left to right the first group of three bars had 19.7 L water day^-1^ and nutrients every other day (10W,1N), the second had 4.9 L water day^-1^ and nutrients every other day (2.5W,1N) and the third had 19.7 L water day^-1^ and nutrients every day (10W,2N). Leftmost bar in each group is Bonus with crystals on all organs, center is *cer.c36* lacking crystals on uppermost internodes and leaf sheaths plus lemmas and rightmost is *cer.j59* lacking crystals on leaf blades. Significance; 1, 2 and 3 stars, p < .05, .01 and .001, respectively.


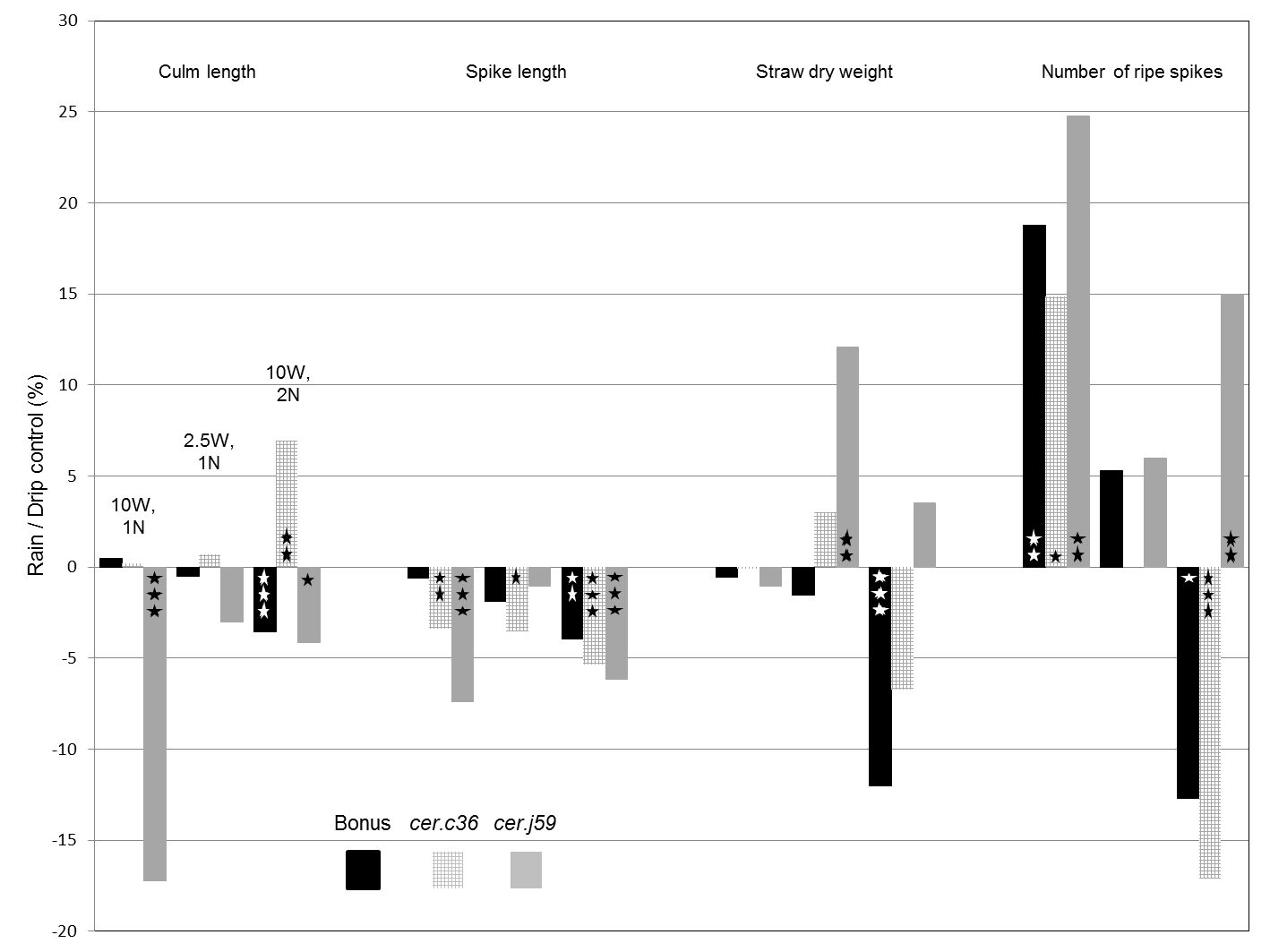


Fig. S4. Effect of rain in the maturation phase on growth traits. From left to right in each panel: Bonus with crystals on all organs, *cer*.*u69* with modified crystals on uppermost internodes and leaf sheaths plus lemmas, .*c36* lacking crystals on uppermost internodes and leaf sheaths plus lemmas plus .*i16* and .*e8* with reductions of crystals on lemmas. All plants received 19.7 L water day^-1^ and nutrients every other day (10W,1N). Significance; 1, 2 and 3 stars, p < .05, .01 and .001, respectively.


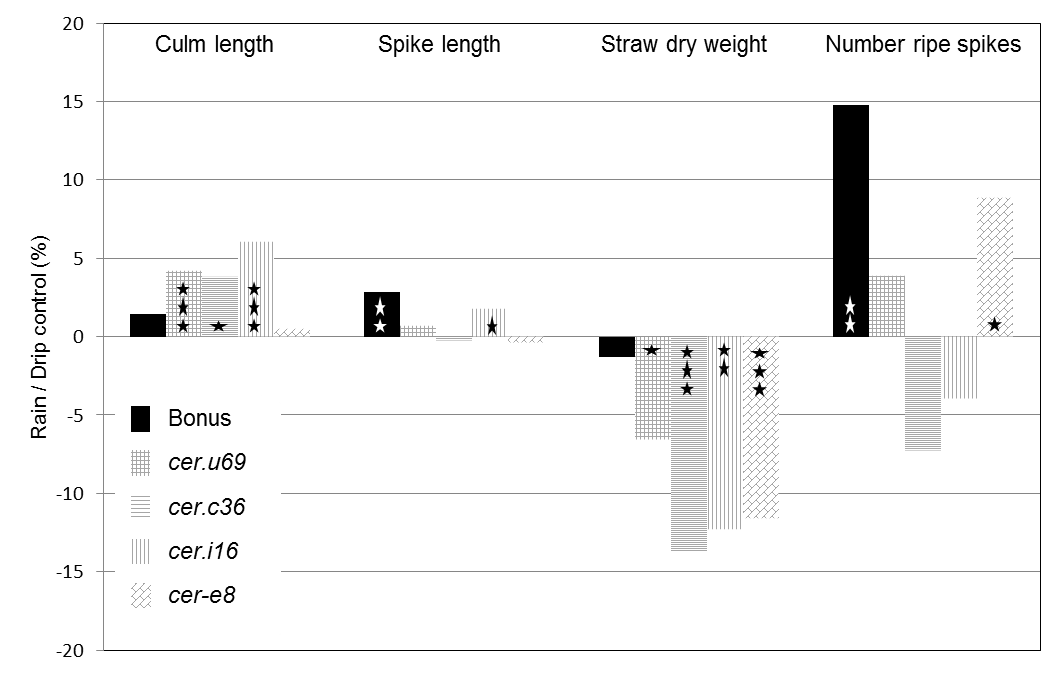


| Table S1. Liters of water per day running through pots on drip and/or rain trucks of Bonus, *cer.c36*, *cer.j59*, *cer.u69*, *cer.i16* or *cer.e8* subjected to different watering^a^ and nutrient^b^ regimes. | | | | | | | | | |
| --- | --- | --- | --- | --- | --- | --- | --- | --- | --- |
| Water during vegetative phase | | | | | |  | Water during heading phase | | |
|  |  | 10W, 1N, 76 days | 2.5W, 1N, 73 days | 10W, 2N, 73 days | 1W, 1N, 73 days |  |  |  | 10W, 1N, 42 days |
| Bonus | Drip | nm | nm | 8.13 | 0.60 |  | Bonus | Rain | 17.75 |
|  | Rain | 10.10 | 2.54 | 9.50 | 1.19 |  |  |  |  |
|  |  |  |  |  |  |  | *cer.u69* | Rain | 18.88 |
| *cer.c36* | Drip | nm | nm | 8.53 | 0.89 |  |  |  |  |
|  | Rain | 11.11 | 2.64 | 9.44 | 1.45 |  | *cer.c36* | Rain | 18.87 |
|  |  |  |  |  |  |  |  |  |  |
| *cer.j59* | Drip | nm | nm | 8.49 | 0.89 |  | *cer.i16* | Rain | 16.94 |
|  | Rain | 10.86 | 2.82 | 10.93 | 1.55 |  |  |  |  |
|  |  |  |  |  |  |  | *cer.e8* | Rain | 17.64 |

^a^10W, 2.5 W and 1W; 19.7, 4.9 and 2.0 L water day^-1^: ^b^1N, nutrients every other day; 2N, nutrients every day; nm, not measured.

| Table S2. Vegetative and yield characteristics of Bonus, *cer.c36* and *.j59* resulting from standard phytotron watering with different amounts of nutrients^a^ and time of shift to the thermoperiod for maturation (average of 14 plants ± SE). | | | | | | | | |
| --- | --- | --- | --- | --- | --- | --- | --- | --- |
|  | **Genotype** | **Culm length (mm)** | **Spike length (mm)** | **Straw dry weight (g)** | **Spike number** | **Kernel number** | **Kernel weight (g)** | **1000 kernel weight (g)** |
| Expt 1 1N, 48 days | Bonus | 786 ± 8 | 216 ± 2 | 8.3 ± 0.2 | 13.8 ± 0.4 | 182 ± 6 | 9.3 ± 0.2 | 51. ± 0.8 |
|  | *cer.c36* | 758 ± 9 | *225 ± 2* | 8.7 ± 0.2 | 15.6 ± 0.6 | **148 ± 5** | **8.0 ± 0.2** | 54.0 ± 0.6 |
|  | *cer.j59* | **733 ± 6** | **190 ± 3** | *7.7 ± 0.1* | **17.3 ± 0.6** | **212 ± 5** | 9.8 ± 0.2 | **46.3 ± 0.7** |
| Expt 2 1N, 59 days | Bonus | 746 ± 7 | 217 ± 3 | 6.7 ± 0.1 | 14.1 ± 0.3 | 204 ± 5 | 10.4 ± 0.2 | 51.2 ± 0.7 |
|  | *cer.c36* | 717 ± 7 | 222 ± 2 | 7.5 ± 0.3 | 15.9 ± 0.7 | *182 ± 5* | 9.2 ± 0.3 | 49.9 ± 1.0 |
|  | *cer.j59* | 698 ± 9 | **193 ± 2** | 6.8 ± 0.1 | *16.8 ± 0.6* | 222 ± 8 | 10.3 ± 0.4 | **46.6 ± 0.9** |
| Expt 3 2N, 60 days | Bonus | 995 ± 22 | 221 ± 2 | 15.6 ± 0.3 | 16.9 ± 0.5 | 298± 8 | 16.1 ± 0.4 | 53.6 ± 0.5 |
|  | *cer.c36* | **938 ± 10** | **231 ± 1** | 14.5 ± 0.6 | 16.8 ± 0.5 | *253± 9* | **12.8 ± 0.5** | **51.2 ± 0.5** |
|  | *cer.j59* | **903 ± 12** | **206 ± 2** | 16.8 ± 0.3 | **20.4 ± 0.9** | **326 ± 7** | 16.6 ± 0.3 | **49.6 ± 0.6** |

^a^1N, nutrients every other day; 2N, nutrients every day. Result of T-test with pertinent Bonus wild type: * p < 0.05**, ****** *p < 0.01*, ***** p < .001**. Yellow, mutant specific phenotype.

| Table S3. Vegetative and yield characteristics of Bonus, *cer.c36* and *.j59* resulting from raining throughout the entire vegetative phase under different regimes of water^a^ and nutrients^b^ (average of 14 plants ± SE). | | | | | | | | |
| --- | --- | --- | --- | --- | --- | --- | --- | --- |
|  | **Genotype** | **Culm length (mm)** | **Spike length (mm)** | **Straw dry weight (g)** | **Spike number** | **Kernel number** | **Kernel weight (g)** | **1000 kernel weight (g)** |
| Expt 1 10W, 1N | Bonus | 764 ± 10 | 203 ± 2 | 6.4 ± 0.2 | 15.6 ± 0.8 | 130 ± 6 | 5.5 ± 0.2 | 44.0 ± 1.4 |
|  | *cer.c36* | 735 ± 12 | **215 ± 2** | **7.9 ± 0.3** | 17.1 ± 0.7 | **91 ± 5** | **4.3 ± 0.2** | 47.9 ± 1.1 |
|  | *cer.j59* | **595 ± 6** | **175 ± 1** | 6.7 ± 0.2 | **19.4 ± 0.6** | 114 ± 5 | *4.3 ± 0.2* | *39.1 ± 0.6* |
| Expt 2 2.5W, 1N | Bonus | 755 ± 6 | 211 ± 2 | 6.4 ± 0.2 | 13.9 ± 0.4 | 164 ± 4 | 7.4 ± 0.2 | 45.3 ± 1.0 |
|  | *cer.c36* | 707 ± 7 | 218 ± 3 | 6.9 ± 0.2 | 14.4 ± 0.7 | *145 ± 5* | *6.6 ± 0.2* | 46.0 ± 0.8 |
|  | *cer.j59* | *678 ± 9* | **185 ± 2** | 6.5 ± 0.1 | 16.0 ± 0.6 | 172 ± 7 | 7.1 ± 0.3 | **41.0 ± 0.7** |
| Expt 3 10W, 2N | Bonus | 921 ± 14 | 218 ± 2 | 11.7 ± 0.3 | 21.4 ± 0.9 | 310 ± 12 | 15.5 ± 0.5 | 50.0 ± 0.9 |
|  | *cer.c36* | **875 ± 11** | **226 ± 1** | 13.2 ± 0.4 | **17.3 ± 0.6** | **202 ± 8** | **10.9 ± 0.4** | **54.8 ± 0.6** |
|  | *cer.j59* | **844 ± 10** | **194 ± 1** | 12.9 ± 0.3 | **28.6 ± 0.6** | **382 ± 13** | 15.5 ± 0.5 | **40.3 ± 0.9** |

^a^10W and 2.5W; 19.7 and 4.9 L water day^-1^. ^b^1N, nutrients every other day; 2N, nutrients every day. Result of T-test with pertinent Bonus wild type: * p < 0.05**, ****** *p < 0.01*, ***** p < .001**. Yellow, mutant specific phenotype.

| Table S4**.** Vegetative and yield characteristics of Bonus, *cer.c36* and *.j59* resulting from drip watering throughout the entire vegetative phase under different regimes of water^a^ and nutrients^b^ (average of 14 plants ± SE). | | | | | | | | |
| --- | --- | --- | --- | --- | --- | --- | --- | --- |
|  | **Genotype** | **Culm length (mm)** | **Spike length (mm)** | **Straw dry weight (g)** | **Spike number** | **Kernel number** | **Kernel weight (g)** | **1000 kernel weight (g)** |
| Expt 1 10W, 1N | Bonus | 760 ± 10 | 204 ± 3 | 6.4 ± 0.2 | 12.6 ± 0.6 | 154 ± 5 | 7.8 ± 0.2 | 51.0 ± 1.4 |
|  | *cer.c36* | 732 ± 9 | **222 ± 2** | **7.9 ± 0.3** | 14.9 ± 0.7 | **123 ± 3** | **6.7 ± 0.2** | 55.1 ± 0.7 |
|  | *cer.j59* | *722 ± 10* | **189 ± 1** | 6.8 ± 0.2 | 15.6 ± 1.0 | 169 ± 3 | 7.6 ± 0.3 | *44.9 ± 1.3* |
| Expt 2 2.5W, 1N | Bonus | 759 ± 8 | 215 ± 2 | 6.5 ± 0.3 | 13.2 ± 0.6 | 194 ± 6 | 8.8 ± 0.3 | 45.7 ± 1.0 |
|  | *cer.c36* | *702 ± 9* | *226 ± 2* | 6.7 ± 0.2 | 14.4 ± 0.6 | *167 ± 5* | 7.8 ± 0.2 | 46.6 ± 0.9 |
|  | *cer.j59* | 699 ± 10 | **187 ± 2** | 5.8 ± 0.2 | 15.1 ± 0.7 | 184 ± 6 | *7.8 ± 0.2* | 42.5 ± 0.8 |
| Expt 3 10W, 2N | Bonus | 955 ± 11 | 227 ± 2 | 13.3 ± 0.2 | 24.4 ± 1.0 | 414 ± 11 | 20.8 ± 0.4 | 50.4 ± 0.7 |
|  | *cer.c36* | **818 ± 11** | **239 ± 2** | 14.2 ± 0.4 | *20.9 ± 0.6* | **297 ± 12** | **17.0 ± 0.7** | **57.1 ± 0.5** |
|  | *cer.j59* | **881 ± 14** | **207 ± 1** | 12.5 ± 0.3 | 24.0 ± 1.2 | 418 ± 16 | 19.4 ± 0.6 | *46.8 ± 1.0* |

^a^10W and 2.5W; 19.7 and 4.9 L water day^-1^. ^b^1N, nutrients every other day; 2N, nutrients every day. Result of T-test with pertinent Bonus wild type: * p < 0.05**, ****** *p < 0.01*, ***** p < .001**. Yellow, mutant specific phenotype.

Table S5. Composition of five wax classes from spikes of Bonus and its mutants *cer-i16* and *.e8*. Hydrocarbons^a^ include alkanes and monoalkenes (=) plus other minor homologous series [Plant Journal 49: 250-264 (2007)]. Esters^b^ consist of those with odd chain secondary alcohols derived from the DKS polyketide pathway, and even chain primary alcohols derived from the KCS acyl pathway.

| Hydrocarbons | | | |  | Esters | | | |  |  |  |  |  |
| --- | --- | --- | --- | --- | --- | --- | --- | --- | --- | --- | --- | --- | --- |
| C no | Bonus | *cer.i16* | *cer.e8* |  | C no | Bonus | *cer.i16* | *cer.e8* |  |  |  |  |  |
| 21 | 0.3 | 0.6 | 3 |  | 31 | 0.7 | tr | tr |  |  |  |  |  |
| 22 | 0.2 | tr | 0.4 |  | 33 | 11.2 | 4.2 | 4.5 |  |  |  |  |  |
| 23 | 1.5 | 2.7 | 9.9 |  | 35 | 16.9 | 7.9 | 7.5 |  |  |  |  |  |
| 24 | 0.2 | 0.1 | 0.6 |  | 36+37 | 3.2 | 1.3 | 0.3 |  |  |  |  |  |
| 25= | tr | 0.1 | 1.5 |  | 38 | 2.1 | 3.5 | 3.5 |  |  |  |  |  |
| 25 | 2 | 3.2 | 10.3 |  | 39 | tr | 0.1 | 0.1 |  |  |  |  |  |
| 26 | 0.1 | 0.1 | 0.5 |  | 40 | 5.7 | 7.5 | 9.3 |  |  |  |  |  |
| 27= | 0.1 | 0.2 | 2.3 |  | 41 | tr | 0.6 | 0.7 |  |  |  |  |  |
| 27 | 2 | 2.8 | 8 |  | 42 | 12.6 | 18.5 | 26.8 |  |  |  |  |  |
| 28 | 0.2 | 0.6 | 0.8 |  | 43 | tr | 0.5 | 0.5 |  |  |  |  |  |
| 29= | 0.1 | 0.4 | 5.5 |  | 44 | 14.9 | 21.3 | 24.8 |  |  |  |  |  |
| 29 | 14.7 | 17.7 | 16.8 |  | 46 | 17.4 | 21.2 | 16.2 |  |  |  |  |  |
| 30 | 0.8 | 0.7 | 4.5 |  | 48 | 7.4 | 7.7 | 4.7 |  |  |  |  |  |
| 31= | tr | 1.1 | 2 |  | 50 | 4.9 | 3.9 | 1.1 |  |  |  |  |  |
| 31 | 72.1 | 65.4 | 29.6 |  | 52 | 3 | 1.6 | 0.1 |  |  |  |  |  |
| 32 | 1 | 1.1 | 1 |  |  |  |  |  |  | Free fatty acids | | | |
| 33= | tr | 0.5 | 1 |  |  |  |  |  |  | C no | Bonus | *cer.i16* | *cer.e8* |
| 33 | 4.1 | 3 | 2.1 |  |  |  |  |  |  | 16 | 4.8 | 7.4 | 7.1 |
|  |  |  |  |  |  |  |  |  |  | 17 | tr | tr | 0.1 |
|  |  |  |  |  |  |  |  |  |  | unk | tr | 2.7 | 0.2 |
| Primary alcohols | | | |  | Aldehydes | | | |  | 18 | 3.7 | 3.6 | 3.5 |
| C no | Bonus | *cer.i16* | *cer.e8* |  | C no | Bonus | *cer.i16* | *cer.e8* |  | 19 | tr | tr | 0.3 |
| 20 | nd | nd | tr |  | 20 | tr | nd | 1.8 |  | 20 | 10.9 | 13.9 | 14.7 |
| 21 | nd | nd | tr |  | 21 | nd | nd | tr |  | 21 | 0.3 | 0.3 | 0.5 |
| 22 | 1.5 | 1.9 | 3.4 |  | 22 | 0.1 | 0.3 | 3.5 |  | 22 | 17 | 17.5 | 29.8 |
| 23 | tr | 0.2 | 0.1 |  | 23 | nd | tr | 0.4 |  | 23 | 0.5 | 0.6 | 1.1 |
| 24 | 6.5 | 8.5 | 9.5 |  | 24 | 0.9 | 1.2 | 2.3 |  | 24 | 13.9 | 15 | 13.9 |
| 25 | 0.5 | tr | tr |  | 25 | nd | tr | 1.9 |  | 25 | 0.3 | tr | tr |
| 26 | 50.8 | 44.4 | 51.8 |  | 26 | 4.1 | 9.4 | 7.4 |  | 26 | 10.2 | 9.7 | 6.5 |
| 27 | 0.6 | tr | tr |  | 27 | 0.2 | 1.1 | 2.7 |  | 27 | 0.2 | tr | tr |
| 28 | 16.4 | 16.8 | 15.8 |  | 28 | 8.4 | 16.8 | 12.4 |  | 28 | 17.2 | 13.5 | 10.4 |
| 29 | 1.1 | 1.7 | 0.8 |  | 29 | 0.5 | tr | 2.3 |  | 29 | 0.7 | 1 | 0.5 |
| 30 | 12 | 13.4 | 10.2 |  | 30 | 27 | 31.9 | 24.7 |  | 30 | 13 | 9.3 | 7.5 |
| 31 | 1.2 | tr | 1.1 |  | 31 | 1.9 | tr | 3.3 |  | 31 | 0.5 | 0.5 | 0.4 |
| 32 | 9.1 | 13.2 | 6.6 |  | 32 | 54.8 | 38.6 | 36 |  | 32 | 6.1 | 4.4 | 2.8 |
| 33 | nd | nd | nd |  | 33 | tr | tr | tr |  | 33 | tr | nd | nd |
| 34 | 0.2 | tr | tr |  | 34 | 2.1 | 0.7 | 1.4 |  | 34 | 0.3 | 0.5 | 0.7 |

C no = carbon number; nd = not detected; tr = trace, <0.1%; Distributions are weight % with alcohols and aldehydes as acetates, and fatty acids as methyl esters. C_36_-1-ol esters and C_37_-2-ol esters were not separated by the GC column used. 1-ol esters with 45, 47, 49 and 51 carbons also occur in trace amounts.

| Table S6. Vegetative and yield characteristics of Bonus, *cer.u69, .c36, .i16* and *.e8* resulting from 10W^a^ watering during the maturation phase under different regimes of nutrients^b^ and water delivery (mean of 14 plants ± SE). | | | | | | | | |
| --- | --- | --- | --- | --- | --- | --- | --- | --- |
|  | **Genotype** | **Culm length (mm)** | **Spike length (mm)** | **Straw dry weight (g)** | **Spike number** | **Kernel number** | **Kernel weight (g)** | **1000 kernel weight (g)** |
| Drip, 1N | Bonus | 836 ± 7 | 211 ± 1 | 6.9 ± 0.1 | 14.0 ± 0.3 | 210 ± 6 | 9.3 ± 0.2 | 43.8 ± 0.6 |
|  | *cer.u69* | 837 ± 6 | 216 ± 1 | 7.3 ± 0.1 | 14.6 ± 0.4 | 198 ± 8 | 9.3 ± 0.3 | 47.5 ± 1.2 |
|  | *cer.c36* | 805 ± 8 | *217 ± 1* | 7.4 ± 0.1 | 14.9 ± 0.5 | **172 ± 5** | **7.5 ± 0.2** | 44.3 ± 0.9 |
|  | *cer.i16* | 815 ± 6 | **202 ± 1** | 7.2 ± 0.1 | 14.6 ± 0.3 | 195 ± 6 | 8.6 ± 0.2 | 44.4 ± 0.7 |
|  | *cer.e8* | 863 ± 6 | 210 ± 1 | 7.1 ± 0.2 | 14.4 ± 0.4 | 214 ± 7 | 9.6 ± 0.3 | 45.1 ± 0.7 |
| Drip, 2N | Bonus | 845 ± 8 | 215 ± 1 | 8.1 ± 0.2 | 18.4 ± 0.6 | 276 ± 8 | 11.3 ± 0.3 | 41.6 ± 0.8 |
|  | *cer.u69* | *875 ± 7* | *218 ± 1* | 7.8 ± 0.1 | 17.3 ± 0.5 | 239 ± 10 | 10.1 ± 0.3 | 42.9 ± 0.9 |
|  | *cer.c36* | *809 ± 7* | 217 ± 2 | 8.2 ± 0.2 | 17.8 ± 0.8 | **175 ± 8** | **7.6 ± 0.2** | 42.3 ± 0.8 |
|  | *cer.i16* | 858 ± 7 | **208 ± 1** | 8.1 ± 0.3 | 17.9 ± 0.7 | 255 ± 9 | 10.3 ± 0.3 | 40.8 ± 0.7 |
|  | *cer.e8* | 873 ± 7 | *218 ± 1* | 8.5 ± 0.2 | 19.0 ± 0.6 | 279 ± 10 | 11.3 ± 0.4 | 40.8 ± 0.9 |
| Rain, 1N | Bonus | 848 ± 5 | 217 ± 1 | 6.8 ± 0.1 | 16.1 ± 0.5 | 234 ± 5 | 7.7 ± 0.2 | 33.1 ± 1.0 |
|  | *cer.u69* | *873 ± 7* | 217 ± 1 | 6.8 ± 0.2 | 15.2 ± 0.3 | 219 ± 7 | 7.9 ± 0.2 | 36.2 ± 0.9 |
|  | *cer.c36* | 836 ±10 | 217 ± 1 | 6.4 ± 0.2 | *13.8 ± 0.5* | **151 ± 5** | **5.7 ± 0.1** | *37.9 ± 0.9* |
|  | *cer.i16* | 865 ± 6 | **206 ± 1** | 6.3 ± 0.1 | 14.1 ± 0.5 | **177 ± 6** | **6.2 ± 0.2** | 35.9 ± 0.7 |
|  | *cer.e8* | 867 ± 6 | **209 ± 1** | *6.3 ± 0.1* | 15.7 ± 0.3 | **196 ± 5** | *7.0 ± 0.2* | 35.7 ± 0.8 |

^a^10W, 19.7 L water day^-1^. ^b^1N, nutrients every other day; 2N, nutrients every day. Result of T-test with Bonus wild type: * p < 0.05**, ****** *p < 0.01*, ***** p < .001**. Yellow, mutant specific phenotype.

| Table S7. Comparison of vegetative (veg) versus maturation (mat) phase data for four Bonus and *cer.c36* phenotypic traits.  A: Delivering 1N and water as drip or rain. Data from Figures 5 and 6 plus Supplementary data Tables S3, S4 and S6. All comparisons have a p < .001, except for the kernel weights of the *cer.c36* drip results that have a p < .01. | | | | | | | | | |
| --- | --- | --- | --- | --- | --- | --- | --- | --- | --- |
|  |  | Culm length  (mm) | | Kernel number | | Kernel wt  (g) | | 1000 kernel weight  (g) | |
|  |  | Veg | Mat | Veg | Mat | Veg | Mat | Veg | Mat |
| Drip | Bonus | 760 ± 10 | 836 ± 7 | 154 ± 5 | 210 ± 6 | 7.8 ± 0.2 | 9.3± 0.2 | 51.0 ± 1.4 | 43.8 ± 0.6 |
|  | *cer.c^36^* | 732 ± 9 | 805 ± 8 | 123 ± 3 | 172 ± 5 | 6.7 ± 0.2 | 7.5 ± 0.2 | 55.1 ± 0.7 | 44.3 ± 0.9 |
|  |  |  |  |  |  |  |  |  |  |
| Rain | Bonus | 764 ± 10 | 848 ± 5 | 130 ±6 | 234 ± 5 | 5.5 ± 0.2 | 7.7 ± 0.2 | 44.0 ± 1.4 | 33.1 ± 1.0 |
|  | *cer.c^36^* | 735 ± 12 | 836 ± 10 | 91 ± 5 | 151 ± 5 | 4.3 ± 0.2 | 5. 7± 0.1 | 47.9 ± 1.1 | 37.9 ± 0.9 |

| B. Delivering 1N and water as drip. Data from Figures 5 and 6 plus Supplementary Tables S4 and S5. All comparisons have a p < .001, except for the culm lengths of the *cer.c36* results that have a p = .51, not significant. | | | | | | | | |
| --- | --- | --- | --- | --- | --- | --- | --- | --- |
|  | Culm length  (mm) | | Kernel number | | Kernel wt  (g) | | 1000 kernel weight  (g) | |
|  | Veg | Mat | Veg | Mat | Veg | Mat | Veg | Mat |
| Bonus | 955 ± 11 | 845 ± 8 | 414 ± 11 | 276 ± 8 | 20.8 ± 0.4 | 11.3 ± 0.3 | 50.4 ± 0.7 | 41.6 ± 0.8 |
| *cer.c^36^* | 818 ± 11 | 809 ± 7 | 297 ±12 | 175 $\pm8$ | 17.0 ± 0.7 | 7.6 ± 0.5 | 57.1 ± 0.5 | 42.3 ± 0..8 |

| Table S8. Crude protein and the dye binding capacity (DBC)^a^ of kernels from Bonus, *cer.c36* and *cer.j59* plants grown under different regimes of 10W^b^ and nutrients^c^ throughout the entire vegetative phase of growth (average of 2 plants). | | | | | | | |
| --- | --- | --- | --- | --- | --- | --- | --- |
|  |  | 1N | | | 2N | | |
|  |  | Bonus | *cer.c36* | *cer.j59* | Bonus | *cer.c36* | *cer.j59* |
| Crude protein (%) | Phytotron standard | 11.1 | 11.8 | 11.6 | 13.8 | 13.3 | 13.6 |
|  | Drip | 11.7 | 11.7 | 12.2 | 12.9 | 13.3 | 12.4 |
|  | Rain | 12.4 | 12.1 | 13.0 | 13.0 | 13.7 | 12.3 |
| DBC | Phytotron standard | 70.9 | 69.1 | 69.3 | 57.7 | 58.6 | 57.1 |
|  | Drip | 69.1 | 69.3 | 68.1 | 61.4 | 59.4 | 61.9 |
|  | Rain | 67.9 | 69.9 | 66.5 | 60.9 | 57.6 | 63.3 |

^a^DBC: µmoles of Acilane Orange G bound / 60 mg crude protein (basic amino acids / 60 mg N x 6.25).

^b^10W, 19.7 L water day^-1^: ^c^1N, nutrients every other day; 2N, nutrients every day.
